# Direct and Allosteric Inhibition of the FGF2/HSPGs/FGFR1 Ternary Complex Formation by an Antiangiogenic, Thrombospondin-1-Mimic Small Molecule

**DOI:** 10.1371/journal.pone.0036990

**Published:** 2012-05-14

**Authors:** Katiuscia Pagano, Rubben Torella, Chiara Foglieni, Antonella Bugatti, Simona Tomaselli, Lucia Zetta, Marco Presta, Marco Rusnati, Giulia Taraboletti, Giorgio Colombo, Laura Ragona

**Affiliations:** 1 Laboratorio NMR, Istituto per lo Studio delle Macromolecole, Consiglio Nazionale delle Ricerche, Milano, Italy; 2 Istituto di Chimica del Riconoscimento Molecolare, Consiglio Nazionale delle Ricerche, Milano, Italy; 3 Department of Oncology, Mario Negri Institute for Pharmacological Research, Bergamo, Italy; 4 Department of Biomedical Sciences and Biotechnology, School of Medicine, University of Brescia, Brescia, Italy; German Research School for Simulation Science, Germany

## Abstract

Fibroblast growth factors (FGFs) are recognized targets for the development of therapies against angiogenesis-driven diseases, including cancer. The formation of a ternary complex with the transmembrane tyrosine kinase receptors (FGFRs), and heparan sulphate proteoglycans (HSPGs) is required for FGF2 pro-angiogenic activity. Here by using a combination of techniques including Nuclear Magnetic Resonance, Molecular Dynamics, Surface Plasmon Resonance and cell-based binding assays we clarify the molecular mechanism of inhibition of an angiostatic small molecule, sm27, mimicking the endogenous inhibitor of angiogenesis, thrombospondin-1. NMR and MD data demonstrate that sm27 engages the heparin-binding site of FGF2 and induces long-range dynamics perturbations along FGF2/FGFR1 interface regions. The functional consequence of the inhibitor binding is an impaired FGF2 interaction with both its receptors, as demonstrated by SPR and cell-based binding assays. We propose that sm27 antiangiogenic activity is based on a twofold–direct and allosteric–mechanism, inhibiting FGF2 binding to both its receptors.

## Introduction

Fibroblast growth factors (FGFs) and their receptors are emerging as promising therapeutic targets for a wide array of pathologies, including angiogenesis-driven diseases. Several human solid tumors, including breast, bladder, prostate, endometrial and lung cancers, as well as haematological malignancies, are associated with deregulated FGF signaling [1]. Aberrant FGF signalling contributes to the development of cancer by acting on both cancer and stromal cells, eliciting different cell functions and biological processes such as angiogenesis and cancer cell proliferation, survival, invasion and metastasis. FGFs signalling requires the formation of a ternary complex composed by FGFs, the high affinity transmembrane tyrosine kinase receptors (FGFR1 through FGFR4), and heparan sulphate proteoglycans (HSPGs) [2]. Therapeutic strategies, aimed at interfering with the formation of the complex between FGF and its receptors (either FGFRs or HSPGs), are being developed and include small molecule inhibitors of FGFR tyrosine kinase activity, monoclonal antibodies targeting FGFRs, and a wide array of natural or synthetic molecules able to sequester FGFs preventing their interaction with FGFRs and HSPGs [3].

**Figure 1:**
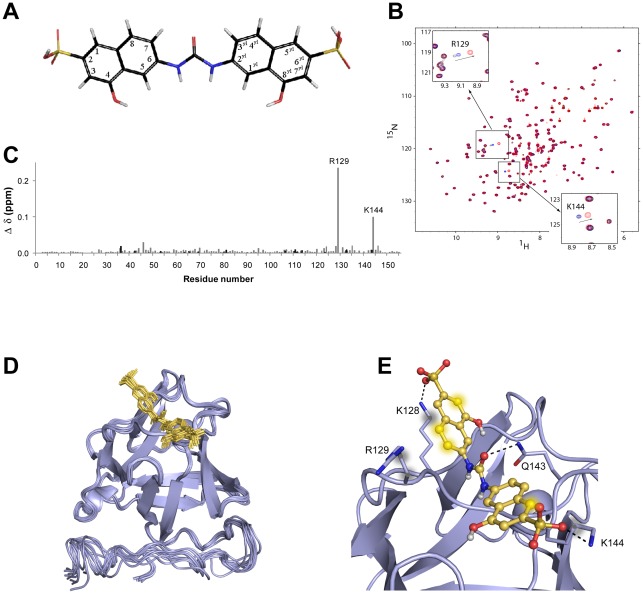
Mapping of FGF2/sm27 interaction by NMR and docking simulations. A) Sm27 molecule: 4-hydroxy-6-[(8-hydroxy-6-sulfonaphthalen-2-yl)carbamoylamino] naphthalene-2-sulfonic acid. B) Superimposed ^1^H-^15^N HSQC spectra of free FGF2 (black), FGF2:sm27 in stoichiometric ratios 1∶1 (blue) and 1∶2 (red). Spectral regions showing R129 and K144 behaviors are zoomed. C) Graphical representation of the combined H^N^ and N FGF2 chemical shift perturbation determined for the various residues, according to [53] (
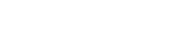
), following the addition of sm27 in 2∶1 stoichiometric ratio. Gray and black bars refer to backbone and Asn, Gln and Arg side chain variations, respectively. D) Bundle of the first 10 structures of cluster 1 obtained with HADDOCK. FGF2 in shown as a blue cartoon, sm27 is shown in yellow sticks. E) Summary of the conserved protein-inhibitor interactions. K128, R129, Q143, and K144 side chains are shown as sticks and are labelled. H-bonds are depicted with black dotted lines and atoms involved in hydrophobic interactions are showed as blurred spheres.

One of the most potent endogenous inhibitors of angiogenesis is thrombospondin-1 (TSP-1) [4], [5]. It binds to FGF2 with an affinity similar to heparin [6], [7], inhibiting the FGF2-mediated angiogenic activation of endothelial cells. We have recently identified an antiangiogenic FGF2-binding site in the type III repeats of TSP-1, and demonstrated that binding of FGF2 to this site inhibits angiogenesis by sequestration of the growth factor [8]. Then, peptide array analysis, binding experiments and SPR analysis guided us to identify a linear amino acidic sequence of type III repeats of TSP-1 that bound FGF2 in the μM range. Using a pharmacophore-based approach, three non-peptidic small molecules, retaining the antiangiogenic activity of the entire TSP-1 and the type III repeats, were identified. The most active molecule, **sm27** (IUPAC name: 4-hydroxy-6-((((8-hydroxy-6-sulfo-2-naphthyl) amino)carbonyl)amino)-2-naphthalenesulfonic acid) (Figure 1A), prevented the binding of FGF2 to endothelial cells, inhibited FGF2-induced endothelial cell proliferation and FGF2-induced angiogenesis in the chicken chorioallantoic membrane assay [9]. Since its stereochemical properties optimally match the design rules proposed to improve the pharmacological applicability of naphthalene sulfonates in antiangiogenesis [10], [11], although with an activity not suitable to make it an immediate drug-candidate, sm27 can be considered the prototype lead compound for the ongoing development of potent FGF2-targeting drugs.

The observed antiangiogenic effects of sm27 could be due to a direct binding of the inhibitor to one of the two distinct binding sites identified for FGFR1 and for heparin/HSPGs [12], [13], [14], [15], [16], [17] or through an indirect perturbation of the conformational properties of the FGF2 sub-structures mostly involved in complex formation with FGF2 receptors. To clarify the molecular details underpinning sm27 inhibition mechanisms, we have set out to characterize the structural properties of the FGF2-sm27 complex and to investigate the perturbative effects of sm27 on the global FGF2 dynamical properties. To this aim, we have used a combination of techniques including Nuclear Magnetic Resonance (NMR) and Molecular Dynamics (MD) simulations. Surface Plasmon Resonance (SPR) and binding assays on cultured cells have been exploited to evaluate the effects of sm27 on FGF2 binding to its two distinct classes of receptors and on the formation of the FGF2/FGFR1/HSPGs ternary complex.

Data discussed herein reveal ligand-dependent modulation of internal motions, suggesting that the non peptidic TSP-1 mimic interferes with the formation of FGF2-receptors complex with a twofold mechanism of action: i) a direct inhibition of FGF2 binding to HSPGs and ii) a long-range modulation of the dynamic properties of the FGF2 surfaces involved FGFR1 recognition.

## Results

### Mapping the FGF/sm27 interaction by NMR and docking simulations

In order to map the sm27-binding site on FGF2, a series of 2D ^15^N-^1^H HSQC experiments were acquired as sm27 was titrated into a solution of ^15^N-enriched FGF2 protein. First, a 19 steps titration was performed from 0∶1 to 4∶1 sm27:FGF2 ratio. The titration progress was suggestive of an intermediate to fast exchange phenomenon, which points to a weak binding. The chemical shift perturbation (CSP) analysis of the FGF2 ^1^H and ^15^N NMR resonances upon addition of sm27 clearly indicated no global tertiary structure modification of the FGF2 molecule upon sm27 binding. Residues showing significant CSP are R129 and K144 (Figure 1B, C), thus clearly identifying the sm27 binding site. This region, located in the long b10–b12 loop, is part of the reported heparin binding site [12], [15], [16], [18], [19]. ^1^H and ^15^N chemical shift deviations were also analyzed separately to finely describe both effects of direct ligand interaction and local structure rearrangements correlating with the binding process [20]. The separate analysis revealed a high variation of R129 ^15^N chemical shift, which was interpreted as a change of K128 side chain conformation upon sm27 binding (Figure S1).

CSP of FGF2 resonances upon sm27 interaction were employed to derive a structural model of the sm27-FGF2 complex through a data driven docking approach with the software HADDOCK2.0 [21], [22]. Ambiguous interaction restraints (AIR) were indeed defined for residues K128, R129 and K144. In addition four unambiguous interaction restraints were defined on the basis of the NOEs observed in the 3D HSQC-NOESY experiment, recorded on the FGF2:sm27 1∶2 sample, .involving R129 and K144 amides and sm27 H5 or H1^yl^ and H7 or H3^yl^ resonances (nomenclature as in Figure 1A, Table S1, Table S2). A total of 4 unambiguous and 18 ambiguous interaction restraints were used to drive the docking process (Table S1). Analysis of the final 200 water-refined models for the FGF2-sm27 complex resulted in 6 different clusters of at least 10 structures and the statistics of the first three clusters is presented in Table S3. All the HADDOCK solutions presented similar features, with sm27 engaging the heparin binding site. Cluster 1 is the most populated and has the best average HADDOCK score and was therefore chosen as representative of the FGF2-sm27 complex (Table S3). The bundle of the 10 lowest-energy structures from cluster 1 was selected for further analysis (Figure 1D). A limited spread within the ensemble was observed, as deduced from RMSD values from their mean structure, calculated on residues 25–155, obtained for Cα: 0.52±0.12 Å and for non-hydrogen atoms: 1.45±0.35 Å. Protein/ligand interactions, conserved in all the ten models, are depicted on the structure in Figure 1E. Two ion-pair electrostatic interactions, involve the cationic side chains of K128 and K144 residues and the anionic sulphonic moiety of sm27. In addition, hydrophobic interactions are established between the carbon atoms of sm27 and the aliphatic branches of K128, R129, Q143, and K144 side chains.

### Characterizing the internal dynamics of FGF2 through NMR

#### Changes in HSQC peak intensities upon sm27 binding

**Figure 2:**
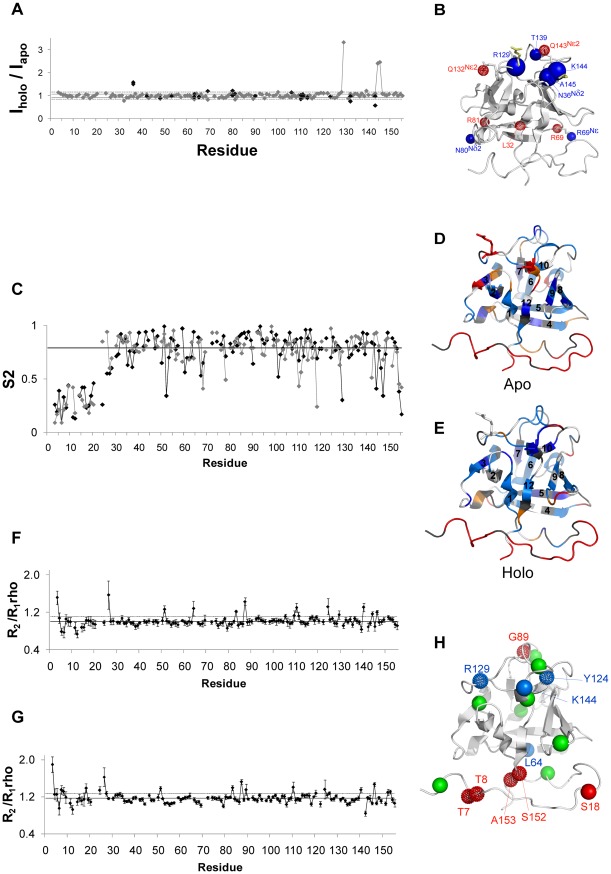
Backbone dynamics of apo and holo FGF2 by NMR. A) ^1^H^15^N-HSQC cross peaks normalized intensity (I) upon sm27 binding vs residue number. Gray and black diamond refers to backbone amides, and glutamine, asparagine and arginine side chains, respectively. B)^ 1^H^15^N-HSQC cross peaks intensity variations plotted on FGF2 molecule. Blue (filled) and red (dotted) spheres indicate residues showing an increased and reduced intensity upon sm27 binding, respectively. The sphere radius is set according to the size of the variation. Side chains affected by intensity variations are shown in sticks. R129 and K144 side chains are shown in yellow sticks for clarity reasons. C) Modelfree analysis order parameters (S2) for the apo- and holo-FGF2 are reported in black and gray, respectively, as a function of the residue number. The black and gray straight lines represent the S2 average value for the apo and holo FGF2, respectively. S2 values are mapped on FGF2 structure in D and E for the apo and holo form, respectively. Color code: red for 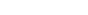, where 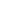 and 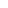
are the average and the standard deviation for S2 values, respectively; orange for 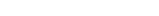; white for 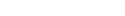; marine for 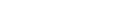; blue for 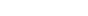. Secondary structure elements are numbered for clarity purposes. F) and G) R2/R1rhovalues as a function of the residue number are reported for the apo and holo FGF2, respectively. The horizontal straight and dotted lines represent the average value and the average plus one standard deviation, respectively. H) Residues with R2/R1rho exceeding from one in the apo and holo FGF2 are mapped on the protein structure. Green spheres represent residues affected by conformational exchange in both the apo and holo form. Marine dotted spheres represent residues affected by conformational exchange only in the apo-FGF2 form. Red spheres represent residues affected by conformational exchange upon sm27 binding. Residues with R2/R1rho values affected by significant errors, either in the apo or in the holo form, are represented as dotted spheres.

Peak intensity in HSQC spectra is a sensitive probe of exchange rates and relaxation rates affecting individual residues. The changes in the normalized peak intensities as a function of ligand additions thus provide useful dynamic and conformational information about the binding event. The peak intensity variations of each FGF2 residue on going from the apo to holo (2∶1 sm27:FGF2 ratio) form is reported in Figure 2A and are summarized on FGF2 structure in Figure 2B. The strongest effects were observed for R129, K144 and A145. Interestingly, these residues showed a very low intensity in the apo-FGF2 HSQC spectrum, probably due to conformational exchange, and their intensities grew up to the average value of the molecule, upon sm27 binding. The behavior of glutamines and asparagine side-chains could be analyzed from the same HSQC spectra, and significant changes were observed for residues N36, Q132 and Q143.

#### T1, T2, and NOE relaxation parameters

Relaxation parameters, R1, R2, and NOE, were measured for the apo and the sm27-bound state of FGF2 to assess the role of protein dynamics, in the picoseconds-nanoseconds time-scale, in the recognition phenomenon.

The comparison of the relaxation parameters obtained for apo and holo FGF2 (Figure S2) indicated a similar overall pattern in the two states. The mobile nature of the long N-terminal tail (residues 3–29) and of the C-terminal tail, suggested by R1, R2 and NOE values is maintained upon sm27 binding. The average R1, R2, and NOE values, calculated excluding the mobile N-terminal tail 1–29 are reported in Table S4. In apo-FGF2, five residues presented R2 values significantly higher than the average plus two standard deviations, namely E87 and G89 (b6–b7 loop), N110 (b8–b9 turn), R129 and K144 (b10–b12 loop). These residues are possibly affected by conformational exchange and/or solvent exchange processes. Interestingly, upon sm27 binding, R129 and K144 R2 values decreased, assuming values typical of the protein core residues, suggesting that the conformational exchange is quenched by the ligand. As reported above, R129 and K144 are the two residues showing the highest chemical shift variation and intensity increase upon sm27 binding, thus defining a sm27 anchoring region.

#### Modelfree analysis

^15^N R1 and R2 relaxation rates and steady-state ^1^H-^15^N NOEs were analyzed using the Modelfree program [23] to obtain values for the residue-by-residue generalized order parameter (S2), which reflects the amplitude of the fast internal motion of the H^N^-N bond vectors in the picoseconds-nanoseconds time scale. The obtained S2 values are reported in Figure 2C as a function of the residue number and plotted accordingly on FGF2 molecule in Figure 2D, E for apo- and holo-FGF2, respectively. Similar average S2 values, calculated for residues 30-153, were obtained for apo (0.79±0.13) and holo-FGF2 (0.78±0.12) indicating that no global conformational flexibility variation occurs upon sm27 binding. The highly flexible nature of the first 29 N-terminal residues was deduced by the very low S2 values for both the apo (S2 = 0.33±0.15) and the holo-FGF2 (0.35±0.24). Likewise, the two C-terminal residues (K154 and S155) presented S2 values lower than 0.5, confirming the considerable disordered nature of these two terminal regions on a picosecond time scale regime. Interestingly, three residues of the N-terminal tail (A11, G24, and F26) drastically reduced their dynamic regime as a consequence of sm27 binding. In particular, G24 and F26 reached S2 values higher than the average.

In apo-FGF2 six residues belonging to the protein core region, namely V52 (b3 strand), E67 (b4 strand), C101 (b7–b8 loop), and R129, K144, L147 (b10–b12 loop), showed a S2 ≤0.53 (corresponding to the average value minus two standard deviations). Upon sm27 binding, motions of all these residues are quenched (Figure 2E). On the other hand, upon sm27 binding, five new residues, namely H59 (b3–b4 loop), N111 (b8–b9 turn), R118 (b9–b10 loop), G142 (b10–b12 loop), and M151 (b12 strand), significantly decreased their S2 to values lower the average.

#### R1rho relaxation parameters

R1rho experiments were run to monitor the possibility that some residues might be affected by slow conformational exchange phenomena in the microsecond-millisecond range in addition to the fast picosecond to nanosecond motions probed by measurements of R1. R2/R1rho ratio values significantly deviating from one are indeed indicative of the presence of conformational exchange. The values of the R2/R1rho ratios, as a function of residue number, obtained for apo- and holo-FGF2 are reported in Figure 2F, G. A number of residues clearly showed ratios exceeding from one in both apo and holo FGF2, namely, E3 and F26 (N-terminal tail), G51 (b3-strand), L83 (b6-strand), E87 (b6–b7 loop), N110 (b8–b9 turn), G140 and I146 (b10–b12 loop) (green spheres in Figure 2H). In the apo form four additional residues were affected by conformational exchange: L64 (b4), Y124 (b10), R129 and K144 (b10–b12 loop) (blue spheres in Figure 2H). Interestingly, upon sm27 binding, L64 and K144 R2/R1rho ratios clearly decreased and got closer to the average, suggesting a quench of conformational exchange. Data relative to residues Y124 and R129, although affected by larger errors, are in the same line. On the other hand, increased conformational exchange upon sm27 binding was observed in the N-terminal (T7, T8, S18) and C-terminal (S152, A153) tails and at the level of b6–b7 loop (G89) (red spheres in Figure 2H).

**Figure 3:**
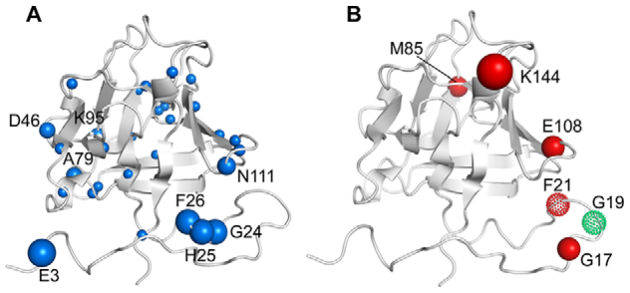
Water mapping by NMR. A) Hydration state of apo-FGF2: residues showing NOE with water higher than the average are represented as spheres with the sphere radius scaled according to the proximity of a water molecule. Serines, threonines, and tyrosines were excluded from the analysis because their peak intensity could be partly due to relayed transfers via the exchanging OH groups of their side-chains. Residues presenting ROE signals are excluded as well. B) Residues affected by NOE and ROE e-PHOGSY intensity variations upon sm27 binding are shown as spheres color coded in red and green, respectively. Filled and dotted spheres indicate the increase and decrease in peak intensity upon sm27 binding, respectively.

#### Mapping protein hydration by NMR

Water molecules at protein surface are critical to their equilibrium structures and to phenomena such as molecular recognition and protein–protein interactions. NMR spectroscopy is a well suited technique to assess the presence of bound waters through ePHOGSY experiments. In this kind of spectra peak intensities depend on distance between water and protein protons, on protein dynamics, and on the residence time of water on the protein [24], [25]. Assuming minor exchange phenomena contribution, ePHOGSY correlations reveal the presence of bound waters at protein sites [24]. Sizeable water-protein Overhauser effects, possibly arising from intermolecular contacts shorter than 4–5 Å between the protein and resident water molecules, were observed for apo and holo protein forms (Figure S3 and S4) at the level of N terminal tail (E3, G24, H25 and F26), in the facing b8–b9 turn (N111) and at the level of residues D46 (b2–b3 loop), A79 (b5–b6 loop), and K95 (b7–b8 loop) (Figure 3A).

Upon sm27 binding a significant increase in intensity of ePHOGSY-NOE correlations occurred in the N-terminal tail and at the level of sm27 binding sites. In particular, residues G17, M85, E108, and K144 were affected (Figure 3B), with major effect observed for K144 (Figure S3 and S4), reflecting an increased residence time and/or a decreased distance of a water molecule from its amide group upon binding. It should be mentioned that G17 presented also a ROE correlation that however didn't change upon sm27 binding. The NOE variation of G17 thus indicated a stabilization of the water molecule close to this residue. At variance, for G19 and F21 a decrease of ROE and NOE effect were observed, respectively.

### Characterizing the internal dynamics of FGF2 through MD simulations

The correlations between structure, dynamics and molecular recognition mechanisms of FGF2 were in parallel investigated by extensive molecular dynamics (MD) simulations on the apo form of FGF2 and on the experimentally derived model of the FGF2/sm27 complex.

From the structural point of view, FGF2 did not undergo any significant conformational change during the course of the simulations: the protein, in both the apo and bound states, visited mainly native conformations, in agreement with NMR observations.

The presence of a specific ligand and its molecular properties, on the other hand, may profoundly affect FGF2 internal dynamics at both the local and global level, even in the absence of major conformational changes.

These dynamic aspects could be characterized by analyzing, in the course of the simulations, the fluctuations of the distances of all pairs of aminoacids in FGF2 and the different patterns originating from the absence/presence of sm27. This analysis, which has been previously developed by some of us [26], [27], [28] was shown to yield information on the existence of rigid/flexible regions in the protein, as well as significant coordination in the dynamics of protein regions that are distant both in physical space and in the primary sequence.

**Figure 4:**
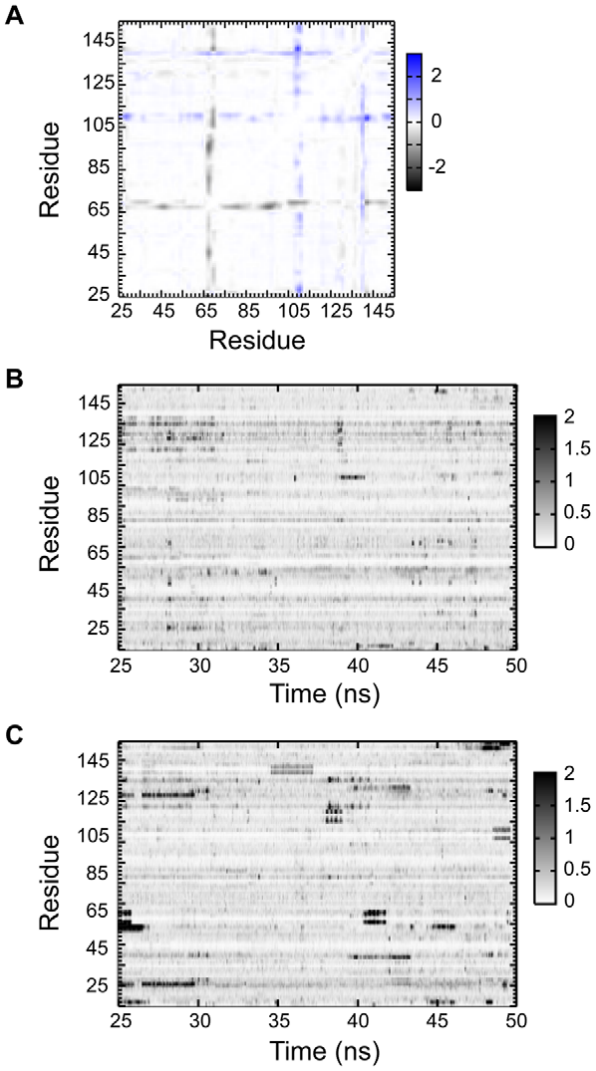
Internal dynamics of apo and holo FGF2. A) Difference matrix obtained by subtracting the distance fluctuations of the apo protein from those of the holo protein. B) Time evolution of the geometrical strain for the apo FGF2; **C.** Time evolution of the geometrical strain for the holo FGF2. The magnitude of the relative fluctuations, expressed in Å^2^ units, is color coded according to the legend on the right of each panel.

The mean squared fluctuations (see Experimental Procedures) of all pair wise distances in the apo and in the simulation of the FGF2/sm27 complex are shown in Figure S5A, B. Due to its high degree of disorder and flexibility, the N-terminal tail was excluded from the analysis and the results refer to the region 25–155. The matrices showed patterns reflecting the alternation of substructures with small and large fluctuations of inter-residue distances. The difference matrix, reporting on the variation of mean squared fluctuations between apo and holo states, is shown in Figure 4A. The presence of the ligand appeared to significantly affect the degree of fluctuations of different groups of FGF2 aminoacids. Specifically, the regions displaying quenched conformational freedom upon binding (represented by gray to black shadings in the matrix) correspond to part of the binding site region (residues 128–131) of b10–b12 loop, and to b4 strand and b4–b5 loop (residues 66–71). Another striking feature of holo protein simulation is the enhancement in the conformational freedom (blue shading in Figure 4A) of residues 139–143 of the binding site, and of residues 107 to 112, corresponding to the b8–b9 turn region. This analysis is unable to catch the differences in the motions observed for loops b3–b4, b7–b8 and b9–b10, identified by NMR analysis. This inconsistency may be due to two main causes: 1) insufficient or limited sampling of the MD-simulations, which limits the conformational statistics that is used for the analysis compared to the experimental situation. This may affect particularly the evaluation of the motional properties of long and flexible loops; 2) the fact that this specific analysis aims at identifying coordinated motions between residue-pairs, while NMR-based indicators mainly refer to single-residue motions. In this framework, in order to analyze the mobility properties of single residues in the context of a dynamic protein environment, we calculated the time-dependent variation of the geometric deformation (also named geometric strain, see [29]) experienced by the various aminoacids in the course of the simulations.

#### Ligand-dependent modulation of strain

We set out to calculate the distortions in the networks of contacts of each residue during the simulations, in the presence or absence of the ligand (see Methods). The parameter that recapitulates the difference between the instantaneous and time-averaged distance of each residue from its neighboring aminoacids is defined as geometric strain. As a consequence, when carried out over the whole trajectory, this quantity provides information on the changes in the local geometry of 3D FGF2 architecture, and provides a compact measure of the local flexibility properties of single residues in the absence and in the presence of the inhibitor. This, in turn, allows for pinpointing specific patterns of structural deformation that emerge in the simulation evolution, providing a direct structural mapping of ligand modulation, which can be qualitatively correlated to NMR-based observations.

Figures 4B, C report the time-dependent geometric strain of each amino acid for the apo and holo simulations analyzed. The analysis showed that in the apo form the only significant strain-related signal emerged in the region centered on residue 107 of b8 strand. In the presence of sm27, a systematic buildup/release of the geometric strain involved specific protein regions, corresponding to residues 34–37 (b1 and b1–b2 loop), 48–52 (b3 strand), 62–67 (b4 strand), 72–74 (b5 strand), and 111–120 (b8–b9 loop, b9 strand, and b9–b10 loop). It is worth noting that the dynamic perturbations of some of these regions were not captured by the analysis of the mean square fluctuations, indicating a possible different nature of the observed effects. Interestingly, consistent changes in the local strain patterns are switched on for the binding region involving residues 127–129, 134–136, and 138–142 of b10–b12 loop.

Finally, the calculation of the average strain experienced by each aminoacid (Figure S6) confirmed the presence of strain hotspots that respond to ligand binding by increasing or decreasing their local flexibility even if they are not in direct contact with sm27. Strain hotspots correspond to the following sequence stretches: 35–39 (b1–b2 loop and b2 strand), 54–57 (b3–b4 loop), 63–67 (b4 strand), 69–72 (b4–b5 turn and b5 strand), 128–132 (b10–b12 loop), and 144 (b10–b12 loop).

### Sm27 inhibition of the interaction of FGF2 with heparin/HSPGs and FGFR1

**Figure 5:**
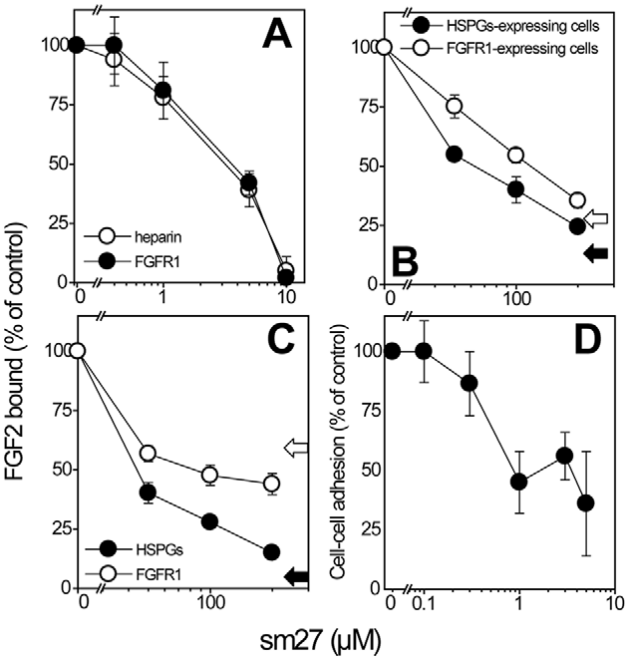
Effect of sm27 on the interaction of FGF2 with heparin/HSPGs and FGFR1. A) FGF2 (150 nM) was injected over heparin or FGFR1 immobilized to a SPR sensorchip in the absence or in the presence of increasing concentrations of sm27 and the amount of FGF2 bound to the surfaces in the different experimental conditions was then measured. Each point is the mean ± SEM of 3 separate experiments. B) Binding of Eu-FGF2 to CHO cells expressing either HSPGs or FGFR1 in the presence of sm27. C) Binding of Eu-FGF2 to endothelial cells (BAEC). D) Effect of sm27 on the formation of the HSPGs/FGF2/FGFR1 complex. FGFR1^+^/HSPG^−^ A745 CHO flg-1A cells were added to a monolayer of FGFR1^−^/HSPG^+^ CHO-K1 cells in serum-free medium with FGF2 (1.66 nM) in the presence of increasing concentrations of sm27. After 2 h of incubation at 298K, the cells bound to the monolayer were counted under an inverted microscope. Each point is the mean ± SEM of 3 separate experiments performed in triplicate. In B and C, black and white arrows indicate the inhibitions obtained with heparin (0.1 µg/ml) and unlabeled FGF2 (1.5 µg/ml) used as reference competitor of HSPGs and FGFR1 binding, respectively

The combined NMR and MD characterization of sm27 binding to FGF2 revealed that the inhibitor targets a well defined region extending over the FGF2 heparin-binding site. We thus exploited SPR analysis to evaluate the ability of sm27 to bind FGF2 and consequently inhibit its interaction with surface-immobilized heparin, an experimental model that resembles the binding of heparin binding growth factors to cell-associated HSPGs [30]. As shown in Figure 5A, sm27 effectively prevents FGF2/heparin interaction in a dose-dependent way with an IC_50_ equal to 3.5 µM, suggesting that the same inhibitory effect can be exerted by sm27 also on the FGF2/HSPGs interaction at the cell surface.

As already described, analysis of the variations in the internal dynamics of the apo- and holo-protein states suggested that sm27 perturbs, through a long-range mechanism, the FGFR1-binding domain of the growth factor (*vide infra*), inferring possible consequences also on the FGF2/FGFR1 interaction. To verify this possibility, SPR was again exploited to evaluate the capacity of sm27 to prevent the binding of FGF2 to substrate-immobilized FGFR1. As shown in Figure 5A and S7, sm27 prevents FGF2/FGFR1 interaction with a potency (ID_50_ equal to 4.0 µM) close to that observed for the inhibition of the FGF2/heparin interaction. Taken together, the above data indicate that sm27 interferes at the same time with the interaction of FGF2 with both HSPGs and FGFR1.

To confirm the interference of sm27 with FGF2 binding to both HSPGs and FGFR1 in a cellular setting, binding of Eu-labelled FGF2 was measured in wild-type CHO-K1 and in FGFR1-transfected A745 CHO flg-1A cells. In these cells, FGF2 binding was entirely mediated by HSPGs in wild-type CHO-K1 cells (expressing HSPGs but not FGFR1) and by FGFR1 in A745 CHO flg-1A cells (deficient in HSPGs and engineered to over-express FGFR1), as confirmed by the inhibitory activity of heparin and unlabelled FGF2 [31] in CHO-K1 and FGFR1-CHO, respectively. In both cells types, FGF2 binding was inhibited by sm27 (Figure 5B), confirming the ability of the small molecule to affect the interaction of FGF2 with both HSPGs and FGFR1. The inhibitory effect of sm27 on FGF2-binding to FGFR1 and HSPGs was confirmed on the more physiological system of endothelial cells, naturally expressing both the classes of receptors. It was possible to discriminate between FGF2 bound to HSPGs low affinity receptors and FGF2 bound to the high affinity FGFR1, following a previously reported procedure [31]. Sm27 inhibited FGF2 binding to both classes of receptors also in this cell system (Figure 5C). In these assays, higher concentrations of sm27 were required to achieve inhibition, possibly because of the higher degree of molecular complexity implied in cell systems, both in terms of variety and concentration of the competing receptors and their interacting ligands.

To exert its pro-angiogenic activity, FGF2 needs to set up a productive, ternary complex with HSPGs and FGFR1 [32]. To investigate whether sm27 was able to prevent the formation of such a complex, a cell-cell adhesion model was used in which FGF2 mediates the interaction of FGFR1^+^/HSPG^−^ A745 CHO flg-1A cells to a monolayer of FGFR1^−^/HSPG^+^ CHO-K1 cells [33]. As shown in Figure 5D, sm27 inhibited FGF2-mediated cell-cell adhesion (ID_50_ equal to 1 μM), thus indicating its capacity to prevent the formation of the FGF2/FGFR1/HSPGs complex. Taken together, all these data are in agreement with our previous observation that sm27 inhibits the mitogenic activity exerted by FGF2 on endothelial cells *in vitro* and FGF2-dependent angiogenesis *in vivo* [9].

## Discussion

The current view of protein structural biology focuses on the fundamental role of dynamic conformational ensembles in molecular recognition [34]. Although there is a large consensus on the role of internal motions in determining function, the understanding, at atomic resolution, of their relationships and relevance in the design/discovery field is more difficult [35], [36].

In this paper, we have used NMR and MD simulations studies to shed light on the variations in the internal motions of FGF2 determined by the binding of the inhibitor sm27, and to propose a direct and allosteric mechanism of inhibition. It was then of pivotal importance the possibility to test this hypothesis through SPR, a cell-free technology that allows the study of macromolecular interactions in well defined conditions, and through more physiological systems represented by binding assays on cultured cells.

NMR and data driven docking analysis clearly indicated that sm27 engages the heparin-binding site of FGF2, making hydrophobic and hydrophilic contacts with residues K128, R129, Q143 and K144. In particular NMR experimental data (CSP and NOE) suggested that residues R129 and K144 are the two hotspots of this interaction. Strong similarities with the inhibitor binding site recently reported for FGF1 [37] are observed. Indeed, the structures of FGF1 in complex with effective inhibitors of angiogenesis (gentisic acid and derivatives, PDB id: 3JUT, 3K1X) identified in the FGF1-corresponding residues (namely K127 and K142) the anchoring points of the inhibitor [37]. The effects of sm27 binding to the heparin binding site were monitored by SPR analysis as well as in cell based binding assays, which demonstrated that sm27 is able to effectively interfere with heparin/FGF2 interaction. In this respect sm27, originally designed to mimic the binding and physiological activity of TSP-1, maintained the properties of TSP-1 and its FGF2-binding sequence [6], [7], [8], [9].

**Figure 6:**
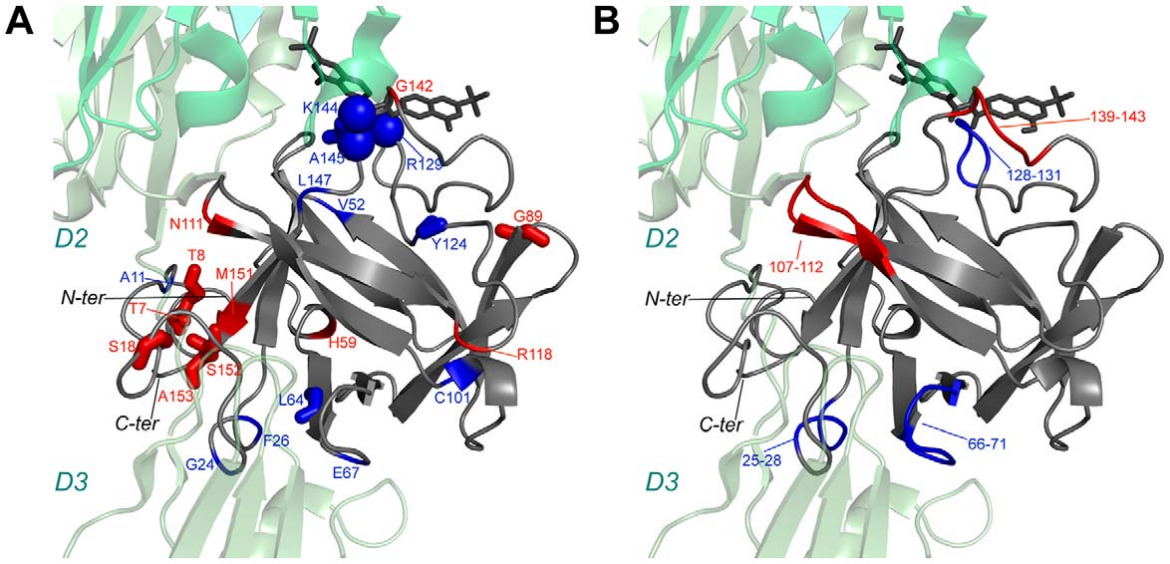
Summary of change in FGF2 dynamics upon sm27 binding . The representative structure of FGF2-sm27 complex is shown in grey. Dynamics variations are mapped on the FGF2 structure. Blu and red colors code for decreased and increased backbone motions upon inhibitor binding. A) NMR data: colored ribbon represents S2 variations, sticks describe slow conformational exchange variations (R_1_rho) and spheres indicate residues affected by HSQC intensity changes upon sm27 binding. B) MD data: mean squared fluctuations differences of all pair wise distances between the apo and holo form. For clarity purposes the complex was superimposed to the X-ray structure of FGFR1-FGF1 dimer (PDB id: 1FQ9) and the FGFR1 regions making contacts with FGF2 are shown. The two FGFR1 units are shown as green and light green cartoons.

Direct interference of sm27 with HSPGs recognition of FGF2 is however only one side of sm27 inhibitory mechanism. Indeed dynamics analysis of the apo and holo FGF2 states performed by NMR and MD, which allows the characterization of motion changes in a timescale range from picosecond to milliseconds, showed that the effects of sm27 binding extended far beyond the interaction site. Figure 6 provides the structural representation of residues affected by dynamical changes upon binding, as deduced by data analysis. Three protein regions could be identified involving residues: i) close to the binding site; ii) distal from the binding site; iii) belonging to N and C-terminal tails. NMR hydration studies detected as well changes of hydration pattern at the level of the binding site and in the flexible N-terminal region upon binding (Figure 3B). Overall, the combined results from NMR and MD investigations revealed common perturbation patterns that impact on functionally relevant regions of FGF2 in response to ligand binding. As shown in the results, the employed MD-derived parameters show variable levels of agreement with NMR-derived data. Specifically, coordination analysis of residue pairs, based on the calculation of mean-square fluctuations, catches motional variations that involve set of residues in part different from the ones revealed by strain analysis. On this basis, we speculate that the observed differences may arise from distinct perturbation mechanisms. The coordination analysis may indicate sets of residues that respond in a cooperative, concerted manner to the presence of the ligand in the binding site. The aminoacids that are highlighted by the strain analysis, on the other hand, may indicate protein regions that respond by simply changing their contacts with the perturbed local environment. At this stage, however, this discussion must be considered as a qualitative one, and further and more quantitative coordination analysis of MD simulations are required [38].

In many biological systems proteins respond to ligand binding by redistributing their internal dynamics [39], [40], [41], [42], [43], which in turn mediate long-range communication. Proteins exist in a number of different substates around their native states, and their relative populations can change following perturbative events, such as ligand binding. In this framework, allostery represents a phenomenon in which a binding event at a certain site perturbs the dynamics of the conformational ensemble, possibly changing the recognition properties at a second site.

In this respect it is worth mentioning that the observed long range effects, detected upon inhibitor binding, affect protein regions directly involved in the tyrosine kinase receptor recognition. Specifically, the perturbed FGF2 regions face the D2, D3 domains and D2-D3 linker of FGFR1[16] (Figure 6). In the same line dynamics variations were observed for FGF1, in the region interacting with the cellular receptor, upon binding of a hexasaccaride heparin-analogue [44].

The functional consequences of the inhibitor induced dynamic perturbation of FGF2 regions involved in receptor binding, could be clearly detected by SPR methods suggesting an impaired FGF2/FGFR1 interaction. Moreover sm27 was able to inhibit FGF2 binding to FGFR1 in two cell systems, the CHO cells, lacking HSPG but expressing FGFR1, and endothelial cells naturally expressing both classes of receptors. The observed perturbations of FGF2/FGFR1 recognition properties thus clearly reported on an allosteric event.

The double action of sm27–direct inhibition of FGF2 binding to heparin and allosteric inhibition of FGF2 binding to FGFR1–has potential profound effects on the activity of the growth factor. The binding of FGF2 to HSPGs is indeed required to set up a productive FGF2/HSPGs/FGFR1 ternary complex that, in turn, allows for FGF2 pro-angiogenic activity [45]. Although other FGF2-antagonist have been described that inhibit the formation of the ternary complex, they always act by specifically interfering with the interaction of the growth factor with either HSPGs [45] or FGFR1 [46], leaving unaffected the other interaction. In this scenario, sm27 represents the first fully characterized example of an FGF2-antagonist that acts by a twofold mechanism of action, i.e. by interfering simultaneously with the two growth factor interactions required for its pro-angiogenic activity.

Because of this unique mechanism of action, sm27 can be considered the prototype of a new class of FGF2-inhibitors. At this stage, we cannot exclude that previously discovered molecules FGF2-directed drugs may also be allosteric modulators, since analyses similar to the ones presented here were not applied. In the context of our investigations, ongoing studies on a set of second generation, sm27-related molecules, will investigate whether it is possible to exploit dynamical information in the design of inhibitors with increased affinity for FGF2 and potentiated inhibition of FGF2/receptor binding and antiangiogenic activity. Assuming that specific interactions underlie the transmission of allosteric mechanisms, a series of sm27-related compounds, in which the original scaffold is decorated with groups that contact residues near the binding site whose dynamic properties have been shown to change, will be tested. MD and NMR evaluation of the long-range effects of such modifications, based on the analysis of perturbed regions and of dynamic coordination pathways, will allow to select profitable allosteric inhibitors. Small molecules causing the same type of long-range perturbation of FGF2 dynamics as observed for sm27 will be selected for further in vitro and cellular analyses of their effects on receptor recognition.

## Materials and Methods

### Chemicals

Sm27 (NSC37204) was from NCI, National Institutes of Health (Rockville, MD). Human recombinant FGF2 was produced and purified as described in Supporting Information S1.

### FGF2/sm27 interactions assessed by NMR

NMR spectra were collected on samples containing 0.57 mM ^15^N-labeled FGF2, dissolved in 50 mM potassium phosphate buffer pH 5.5 (90% H_2_O/10% D_2_O), 2 mM NaN_3_, 10 mM deuterated DTT, in the presence of variable concentrations of sm27 (in the range 0 to 2 mM). All NMR data were collected on a Bruker DMX 500 MHz spectrometer at 298 K.

### Model of the FGF2/sm27 complex

Models of the FGF2/sm27 complex were generated with the data driven docking simulations software HADDOCK2.0 [22] using AIR deduced from CSP data. NOE intermolecular contacts were converted into distances and used as unambiguous restraints. RMSD values of ensemble from their mean structure was calculated with PROFIT (Martin, A. C. R., http://www.bioninf.org.uk/software/profit).

### ^15^N relaxation data acquisition and analysis

HSQC-based experiments were recorded to measure ^15^N longitudinal T1 and transverse T2 relaxation rates, heteronuclear ^1^H→^15^N NOE experiments and ^15^N rotating frame relaxation rates T1rho. Relaxation parameters were analyzed with the program Modelfree 4.20 [23]. The relaxation data were analyzed with both isotropic and axially symmetric model and the latter model was selected. Backbone relaxation data were fit to the five standard Lipari–Szabo model-free formalism models [23].

### NMR Hydration studies

2D ePHOGSY-HSQC experiments with NOE and ROE steps were recorded in order to discriminate between Overhauser and exchange effects. The pulse used for selective water excitation was a 50 ms long 180° Gaussian pulse. The mixing and spin-lock periods for NOE and ROE steps, respectively, were 80 ms long.

### Molecular Dynamics studies of the apo FGF2 and FGF2/sm27 complex

The Apo structure of FGF-2 and the structure of the complex obtained as described above were each subjected to MD simulation analysis to a 100 ns long Molecular Dynamics (MD) simulation in explicit water.

All MD simulations were performed using the AMBER 9.0 package [47] with the ff03 force field, the TIP3P water model [48], and the Particle Mesh Ewald summation method (PME) to deal with long-range Coulomb interactions [49].

### Surface plasmon resonance (SPR) analysis

SPR measurements were performed on a BIAcore X instrument (GE-Healthcare, WI). Heparin was immobilized onto the SPR chip as described [30], allowing the immobilization of 80 resonance units (RU) (6.0 fmol/mm^2^). Immobilization of FGFR (13,800 RU, 42.0 fmol/mm^2^), is described in the Supporting Information S1. Sensorchips coated with streptavidin or FGF-unrelated noggin protein were used for blank subtraction (respectively for heparin and FGFR-1). For competition experiments, FGF2 was injected over the heparin or FGFR1 surfaces for 5 min in the presence of sm27 and washed until dissociation was observed.

### Cell cultures

Wild-type CHO-K1 cells not expressing FGFR1 and the derived HSPG-deficient A745 CHO cell mutants [50] were kindly provided by J. D. Esko (La Jolla, CA). FGFR1-transfected A745 CHO were generated as described in [51]. Cells were grown in Ham's F-12 with 10% FCS. Bovine aortic endothelial cells (BAEC) [52], provided by E. Dejana (Milano, Italy) were cultured in DMEM with 10% FCS.

### Binding of FGF2 to cells

Binding of Europium-labeled FGF2 (Eu-FGF2, 10 ng/ml) to BAEC, CHO-K1 and FGFR1-expressing A745 CHO cells was measured in the presence of sm27, heparin or unlabeled FGF2, essentially as described [9]. Binding to low and high affinity receptors was analyzed according to [31].

### FGF2-mediated cell-cell adhesion assay

FGF-2-dependent binding of A745 CHO flg-1A cells to a monolayer of fixed, wild-type CHO-K1 cells was measured in the presence of increasing concentrations of sm27, as described [33]. Data are mean bound cells in three random microscopic fields.

Full details of all the procedures are given in the Supporting Information S1.

## Supporting Information

Figure S1 **^1^H and ^15^N chemical shift perturbation analysis**. Separate ^1^H and ^15^N analysis of chemical shift perturbation in 2∶1 sm27:FGF2 sample.
(DOC)

Figure S2 **NMR** **relaxation parameters (R1, R2 and ^1^H-^15^N NOE).** R1, R2 and ^1^H-^15^N NOE values measured for apo (left) and holo (FGF2:sm27 1∶2) protein (right) at 500 MHz and 298K are plotted as a function of residue number.
(DOC)

Figure S3 **ePHOGSY spectra of apo and holo forms.** ^1^H-^15^N ePHOGSY HSQC spectra with NOE step for the apo-(A) and holo-FGF2 (B), and with ROE step for the apo (C) and holo-FGF2 (D). In A and B, negative peaks are colored in red. In B and D insets, peaks are colored black and blue for the apo and holo forms, respectively.
(DOC)

Figure S4 **Comparison of ePHOGSY cross peak intensities of apo and holo forms.** ePHOGSY NOE (gray) and ROE (blue) normalized intensities are shown as a function of the residue number in A and B for the apo and holo FGF2, respectively. Straight and dotted lines correspond to the thresholds employed to classify the residues in three groups on the basis of the entity of the observed ePHOGSY effects. In C, the ratio of the normalized intensities observed for holo and apo FGF2 is reported as a function of the residue number for ePHOGSY NOE (gray) and ROE (blue) correlations. Dotted lines delimit intensity variations beyond ± 50% upon sm27 binding. Residues showing significant variations are labeled.
(DOC)

Figure S5 **Distance fluctuation matrices for apo and holo FGF2.** Mean squared fluctuations of all pair wise distances in the apo (A) and in the simulation of the FGF2/sm27 complex (B) The magnitude of pairwise distance fluctuations, expressed in Å^2^ units, is color coded from blue (small fluctuations) to white (large fluctuations).
(DOC)

Figure S6 **Average strain profile.** The residue based profile of average strain calculated over the simulation time for the apo (black line) and for the holo (red line) FGF2.
(DOC)

Figure S7 **SPR analysis of the effect of sm27 on FGF2 interaction with FGFR1 and heparin**. Blank-subtracted sensorgrams showing the binding of FGF2 (150 nM) in the absence (straight lanes) or in the presence (dashed lines) of sm27 (5 nM) to a BIAcore sensorchip coated with heparin (upper panel) or FGFR1 (lower panel). The response (in resonance units, RU) was recorded as a function of time.
(DOC)

Table S1 **Summary of unambiguous and ambiguous interaction restraints employed for HADDOCK calculations.**
(DOC)

Table S2 **^1^H chemical shift of sm27 molecule at pH** **5.5, 298K.**
(DOC)

Table S3 Statistics of the top three FGF2-sm27 clusters obtained with HADDOCK.
(DOC)

Table S4 Average NMR relaxation parameters values of apo- and holo-FGF2 calculated over the residue range 30–152.
(DOC)

Information S1 **Full details of the experimental procedures.**
(DOC)
