# Supplementary material for: Direct and Allosteric Inhibition of the FGF2/HSPGs/FGFR1 Ternary Complex Formation by an Antiangiogenic, Thrombospondin-1-Mimic Small Molecule
Source: PLoS One. 2012 May 14;7(5):e36990. doi: 10.1371/journal.pone.0036990 (PMC3351436; doi:10.1371/journal.pone.0036990)
Supplement: Figure S1 — 1H and 15N chemical shift perturbation analysis. Separate 1H and 15N analysis of chemical shift perturbation in 2∶1 sm27:FGF2 sample. (DOC) [file pone.0036990.s001.doc]

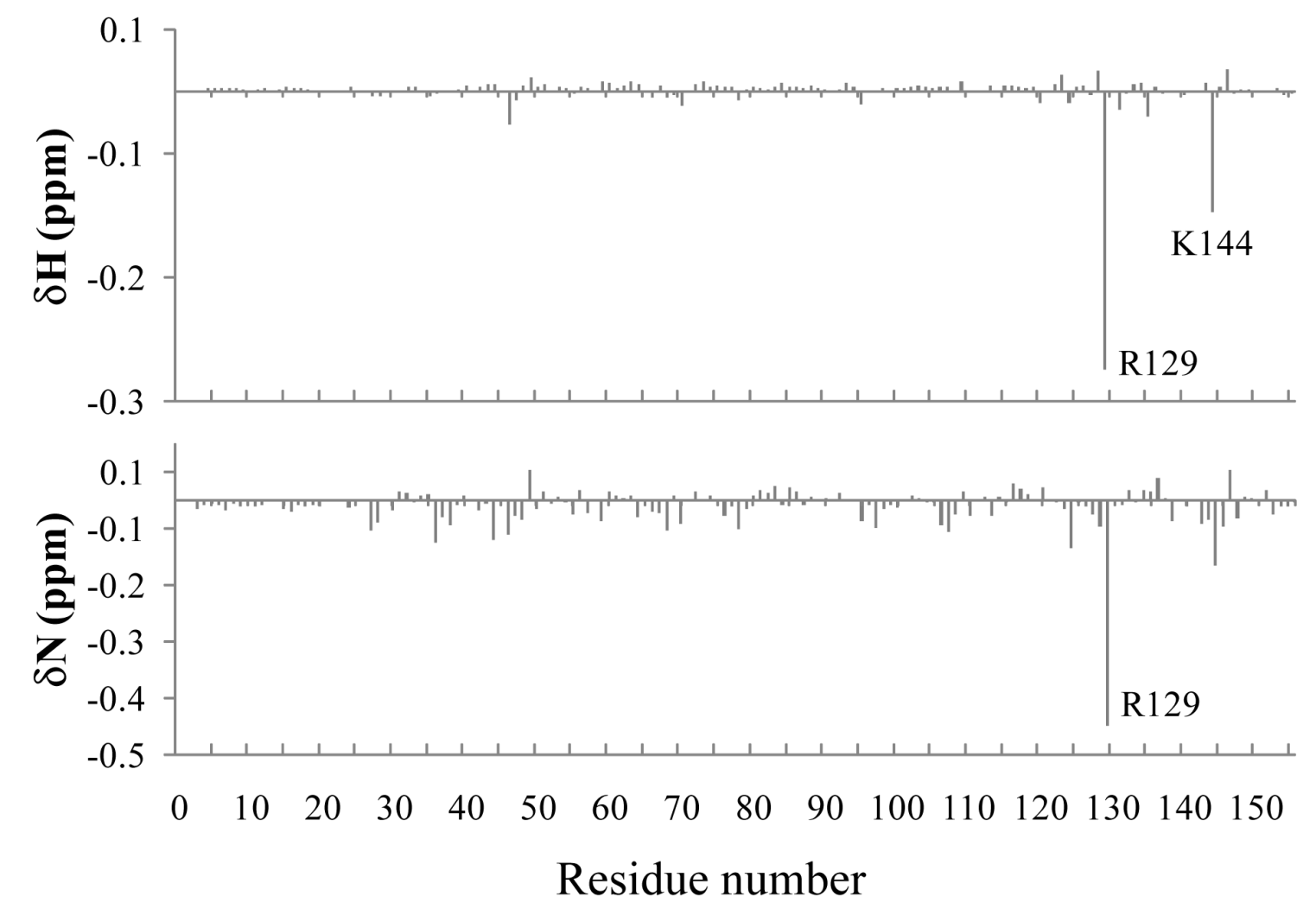


**Figure S1. ^1^H and ^15^N chemical shift perturbation analysis**. Separate ^1^H and ^15^N analysis of chemical shift perturbation in 2:1 sm27:FGF2 sample.
