# Supplementary material for: Direct and Allosteric Inhibition of the FGF2/HSPGs/FGFR1 Ternary Complex Formation by an Antiangiogenic, Thrombospondin-1-Mimic Small Molecule
Source: PLoS One. 2012 May 14;7(5):e36990. doi: 10.1371/journal.pone.0036990 (PMC3351436; doi:10.1371/journal.pone.0036990)
Supplement: Figure S2 — NMR relaxation parameters (R1, R2 and 1H-15N NOE). R1, R2 and 1H-15N NOE values measured for apo (left) and holo (FGF2:sm27 1∶2) protein (right) at 500 MHz and 298K are plotted as a function of residue number. (DOC) [file pone.0036990.s002.doc]

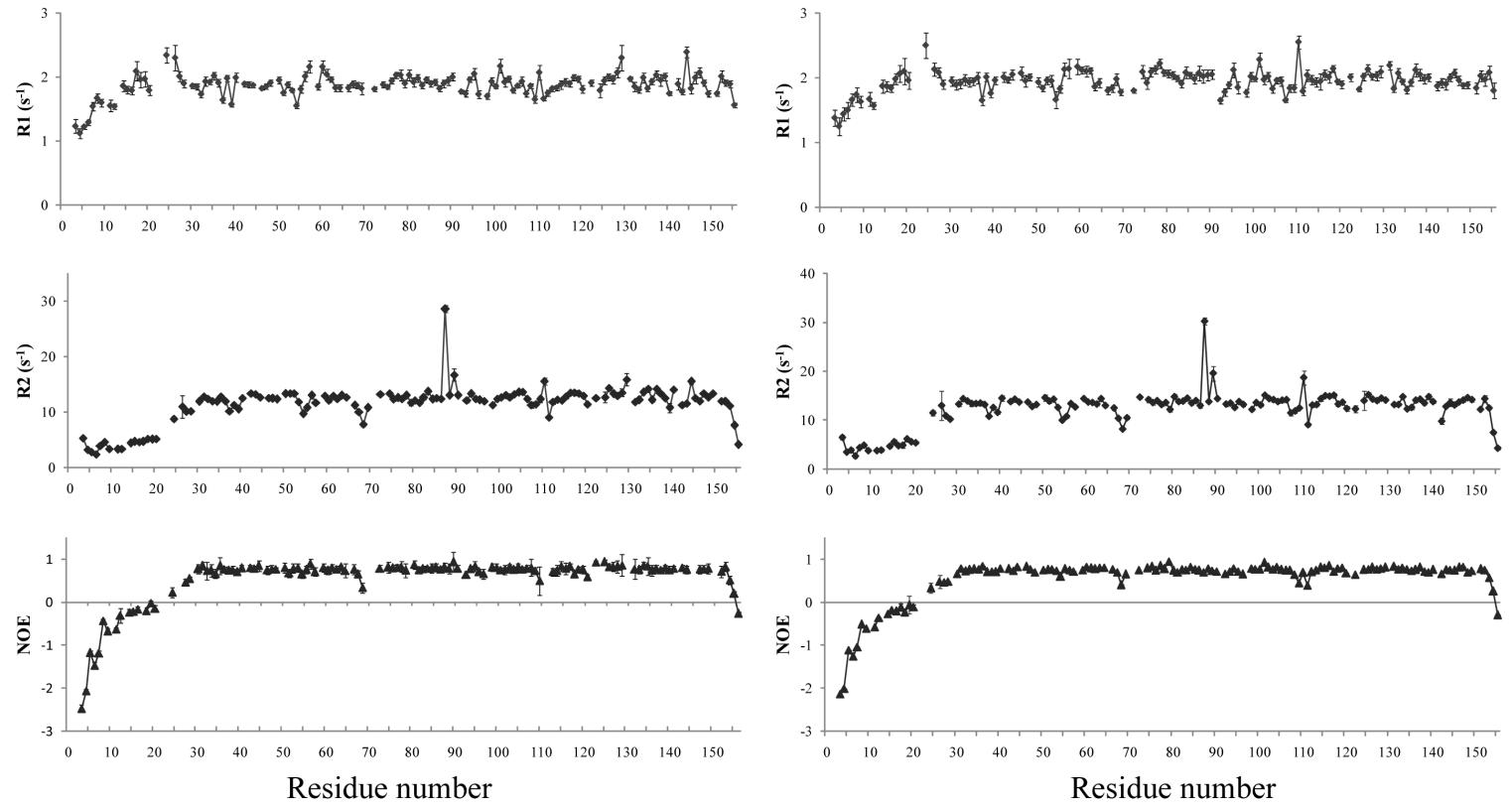


**Figure S2. NMR** **relaxation parameters (R1, R2 and ^1^H-^15^N NOE).** R1, R2 and ^1^H-^15^N NOE values measured for apo (left) and holo (FGF2:sm27 1:2) protein (right) at 500 MHz and 298K are plotted as a function of residue number.
