# Supplementary material for: Direct and Allosteric Inhibition of the FGF2/HSPGs/FGFR1 Ternary Complex Formation by an Antiangiogenic, Thrombospondin-1-Mimic Small Molecule
Source: PLoS One. 2012 May 14;7(5):e36990. doi: 10.1371/journal.pone.0036990 (PMC3351436; doi:10.1371/journal.pone.0036990)
Supplement: Figure S3 — ePHOGSY spectra of apo and holo forms. 1H-15N ePHOGSY HSQC spectra with NOE step for the apo-(A) and holo-FGF2 (B), and with ROE step for the apo (C) and holo-FGF2 (D). In A and B, negative peaks are colored in red. In B and D insets, peaks are colored black and blue for the apo and holo forms, respectively. (DOC) [file pone.0036990.s003.doc]

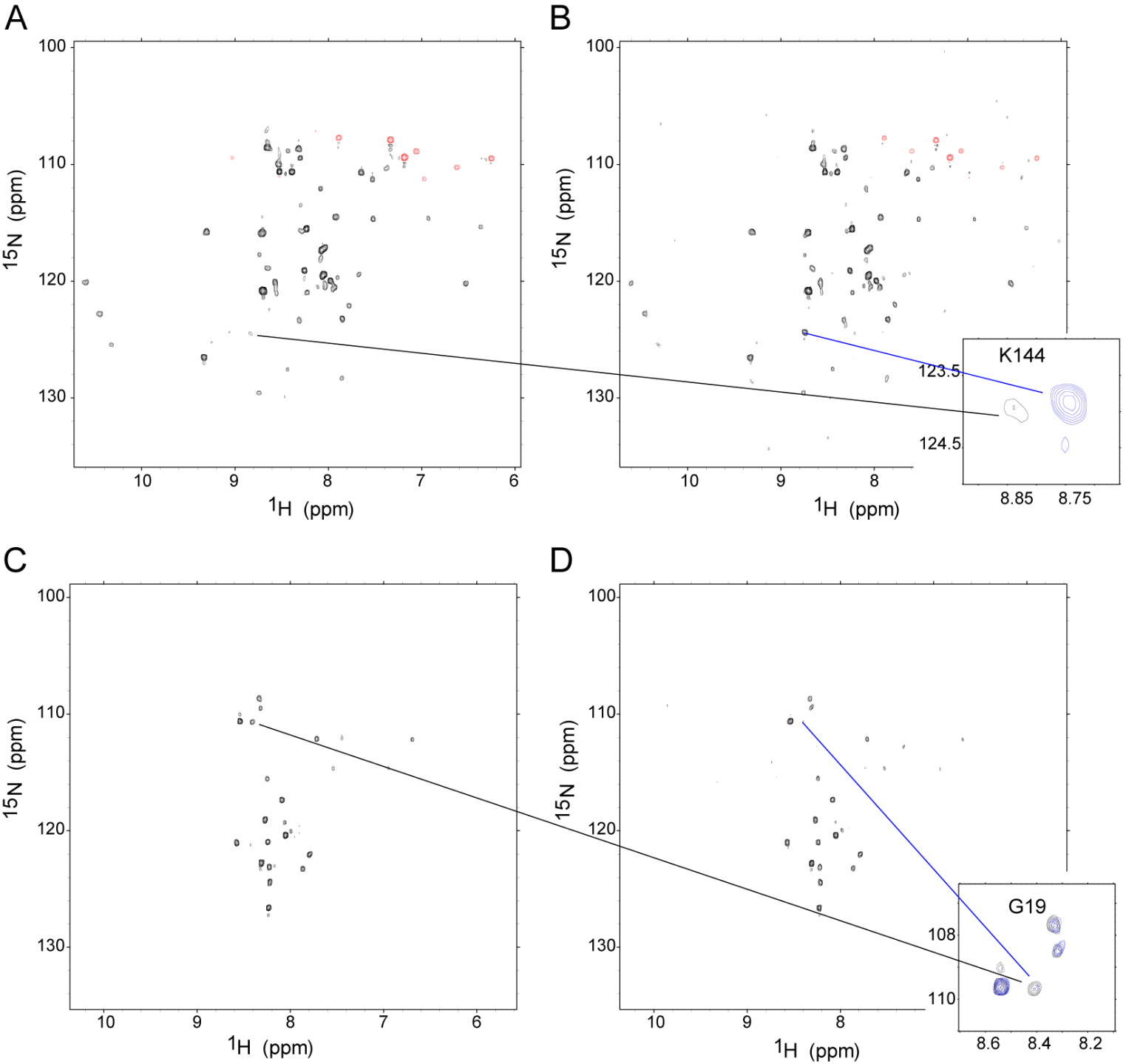
**Figure S3. ePHOGSY spectra of apo and holo forms.** 1H-15N ePHOGSY HSQC spectra with NOE step for the apo-(A) and holo-FGF2 (B), and with ROE step for the apo (C) and holo-FGF2 (D). In A and B, negative peaks are colored in red. In B and D insets, peaks are colored black and blue for the apo and holo forms, respectively.
