# Supplementary material for: Direct and Allosteric Inhibition of the FGF2/HSPGs/FGFR1 Ternary Complex Formation by an Antiangiogenic, Thrombospondin-1-Mimic Small Molecule
Source: PLoS One. 2012 May 14;7(5):e36990. doi: 10.1371/journal.pone.0036990 (PMC3351436; doi:10.1371/journal.pone.0036990)
Supplement: Figure S4 — Comparison of ePHOGSY cross peak intensities of apo and holo forms. ePHOGSY NOE (gray) and ROE (blue) normalized intensities are shown as a function of the residue number in A and B for the apo and holo FGF2, respectively. Straight and dotted lines correspond to the thresholds employed to classify the residues in three groups on the basis of the entity of the observed ePHOGSY effects. In C, the ratio of the normalized intensities observed for holo and apo FGF2 is reported as a function of the residue number for ePHOGSY NOE (gray) and ROE (blue) correlations. Dotted lines delimit intensity variations beyond ± 50% upon sm27 binding. Residues showing significant variations are labeled. (DOC) [file pone.0036990.s004.doc]

**
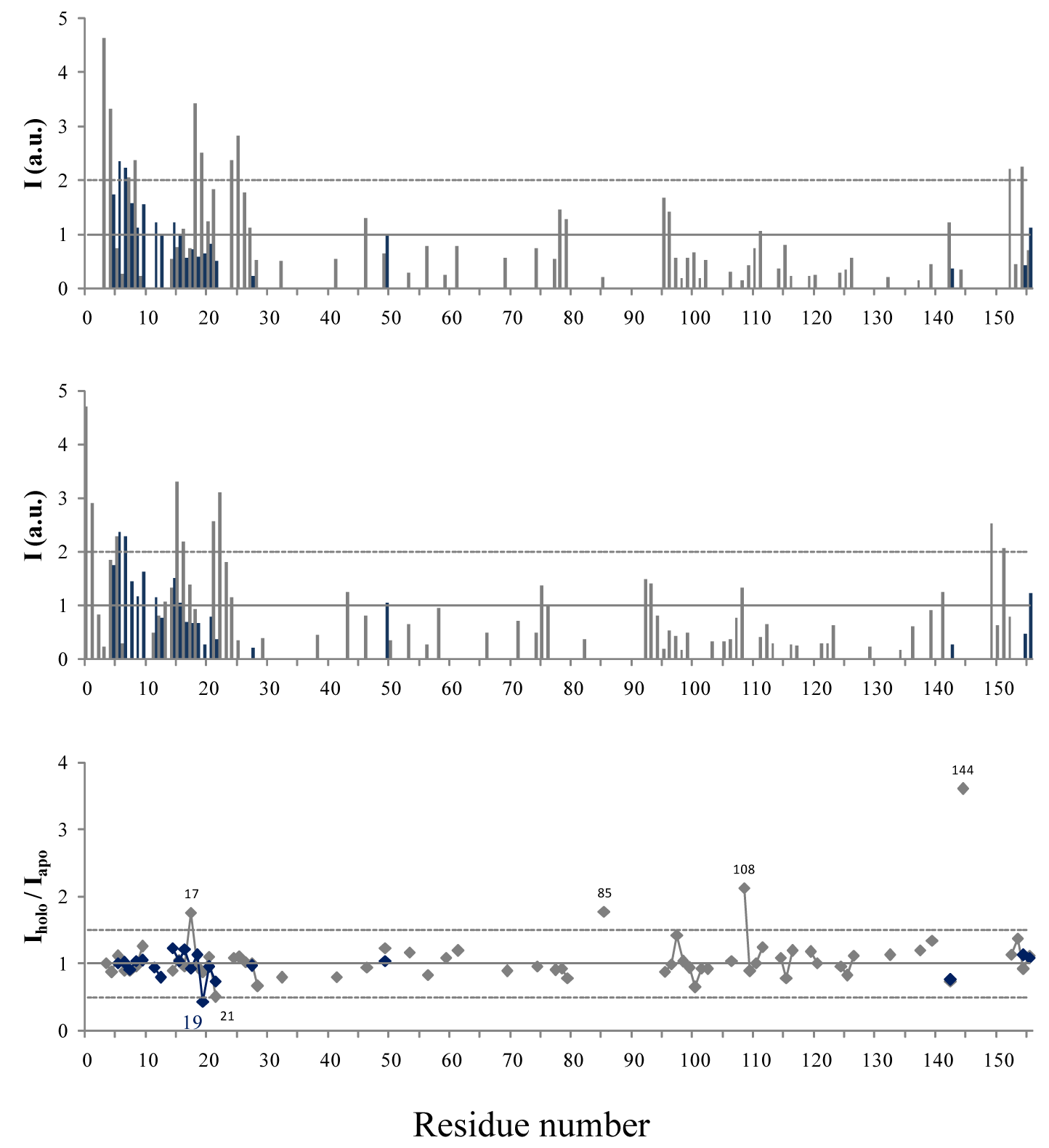
**

**Figure S4. Comparison of ePHOGSY cross peak intensities of apo and holo forms.** ePHOGSY NOE (gray) and ROE (blue) normalized intensities are shown as a function of the residue number in A and B for the apo and holo FGF2, respectively. Straight and dotted lines correspond to the thresholds employed to classify the residues in three groups on the basis of the entity of the observed ePHOGSY effects. In C, the ratio of the normalized intensities observed for holo and apo FGF2 is reported as a function of the residue number for ePHOGSY NOE (gray) and ROE (blue) correlations. Dotted lines delimit intensity variations beyond ± 50% upon sm27 binding. Residues showing significant variations are labeled.
