# Supplementary material for: Direct and Allosteric Inhibition of the FGF2/HSPGs/FGFR1 Ternary Complex Formation by an Antiangiogenic, Thrombospondin-1-Mimic Small Molecule
Source: PLoS One. 2012 May 14;7(5):e36990. doi: 10.1371/journal.pone.0036990 (PMC3351436; doi:10.1371/journal.pone.0036990)
Supplement: Figure S5 — Distance fluctuation matrices for apo and holo FGF2. Mean squared fluctuations of all pair wise distances in the apo (A) and in the simulation of the FGF2/sm27 complex (B) The magnitude of pairwise distance fluctuations, expressed in Å2 units, is color coded from blue (small fluctuations) to white (large fluctuations). (DOC) [file pone.0036990.s005.doc]

**
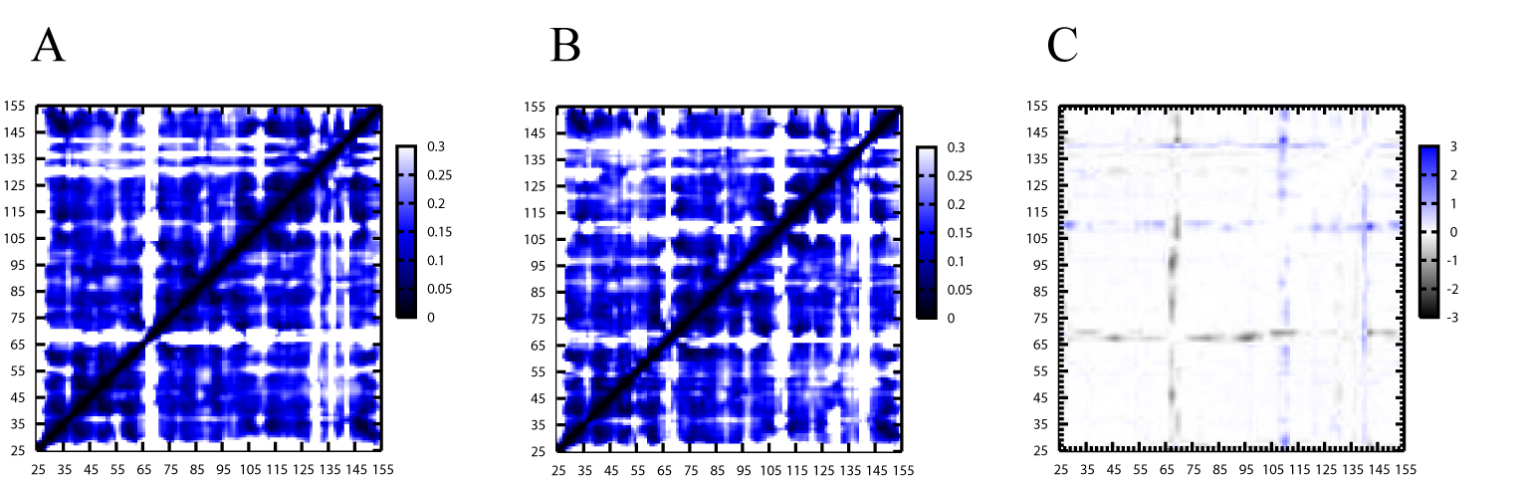
**

**Figure S5. Distance fluctuation matrices for apo and holo FGF2.** Mean squared fluctuations of all pair wise distances in the apo (A) and in the simulation of the FGF2/sm27 complex (B) The magnitude of pairwise distance fluctuations, expressed in Å^2^ units, is color coded from blue (small fluctuations) to white (large fluctuations).
