# Supplementary material for: Direct and Allosteric Inhibition of the FGF2/HSPGs/FGFR1 Ternary Complex Formation by an Antiangiogenic, Thrombospondin-1-Mimic Small Molecule
Source: PLoS One. 2012 May 14;7(5):e36990. doi: 10.1371/journal.pone.0036990 (PMC3351436; doi:10.1371/journal.pone.0036990)
Supplement: Figure S6 — Average strain profile. The residue based profile of average strain calculated over the simulation time for the apo (black line) and for the holo (red line) FGF2. (DOC) [file pone.0036990.s006.doc]

**
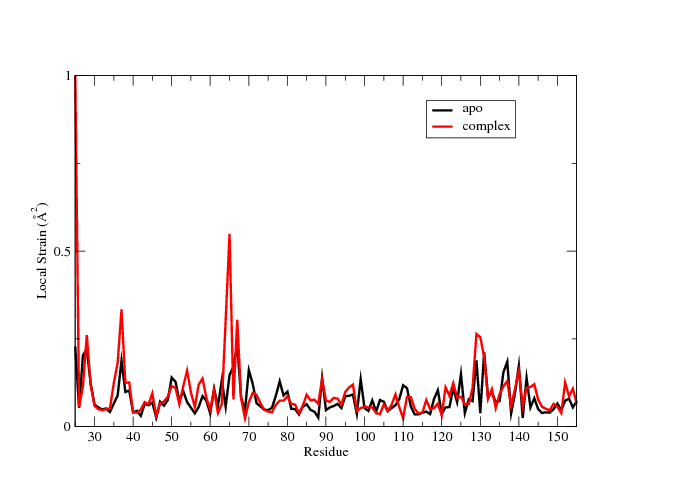
**

**Figure S6. Average strain profile.** The residue based profile of average strain calculated over the simulation time for the apo (black line) and for the holo (red line) FGF2.
