# Supplementary material for: Direct and Allosteric Inhibition of the FGF2/HSPGs/FGFR1 Ternary Complex Formation by an Antiangiogenic, Thrombospondin-1-Mimic Small Molecule
Source: PLoS One. 2012 May 14;7(5):e36990. doi: 10.1371/journal.pone.0036990 (PMC3351436; doi:10.1371/journal.pone.0036990)
Supplement: Figure S7 — SPR analysis of the effect of sm27 on FGF2 interaction with FGFR1 and heparin. Blank-subtracted sensorgrams showing the binding of FGF2 (150 nM) in the absence (straight lanes) or in the presence (dashed lines) of sm27 (5 nM) to a BIAcore sensorchip coated with heparin (upper panel) or FGFR1 (lower panel). The response (in resonance units, RU) was recorded as a function of time. (DOC) [file pone.0036990.s007.doc]

**Figure S7. SPR analysis of the effect of sm27 on FGF2 interaction with FGFR1 and heparin**. Blank-subtracted sensorgrams showing the binding of FGF2 (150 nM) in the absence (straight lanes) or in the presence (dashed lines) of sm27 (5 nM) to a BIAcore sensorchip coated with heparin (upper panel) or FGFR1 (lower panel). The response (in resonance units, RU) was recorded as a function of time.
