# Supplementary material for: Direct and Allosteric Inhibition of the FGF2/HSPGs/FGFR1 Ternary Complex Formation by an Antiangiogenic, Thrombospondin-1-Mimic Small Molecule
Source: PLoS One. 2012 May 14;7(5):e36990. doi: 10.1371/journal.pone.0036990 (PMC3351436; doi:10.1371/journal.pone.0036990)
Supplement: Table S1 — Summary of unambiguous and ambiguous interaction restraints employed for HADDOCK calculations. (DOC) [file pone.0036990.s008.doc]

Table S1. Summary of unambiguous and ambiguous interaction restraints employed for HADDOCK calculations.

| ***Unambiguous (NOE derived)^(a)^*** | **FGF2:Sm27** |
| --- | --- |
|  | R129 H^N^ : (H5 or H1^yl^)^(b)^ |
|  | K144 H^N^ : (H5 or H1^yl^) |
|  | R129 H^N^ : (H7 or H3^yl^) |
|  | K144H^N^ : (H7 or H3^yl^) |
| ***Ambiguous (CSP derived)*** |  |
| Active | K128 side chain, R129, K144 |
| Passive | K35, N36, G37, F39, L127, T130, Q132, Y133, K134, L135, K138, G142, Q143, A145, I146 |

^(a)^An allowance of ±30% was introduced for each distance.

^(b)^It was not possible to distinguish the two proton resonances because of the symmetry of sm27molecule.
