# Supplementary material for: Direct and Allosteric Inhibition of the FGF2/HSPGs/FGFR1 Ternary Complex Formation by an Antiangiogenic, Thrombospondin-1-Mimic Small Molecule
Source: PLoS One. 2012 May 14;7(5):e36990. doi: 10.1371/journal.pone.0036990 (PMC3351436; doi:10.1371/journal.pone.0036990)
Supplement: Table S2 — 1H chemical shift of sm27 molecule at pH 5.5, 298K. (DOC) [file pone.0036990.s009.doc]

**Table S2.** **^1^H chemical shift of sm27 molecule at pH 5.5, 298K.**

| **Proton ^(a)^** | **δ(ppm)** |
| --- | --- |
| H^N^ | 8.813 |
| 1 | 7.807 |
| 3 | 7.153 |
| 5 | 8.127 |
| 7 | 7.586 |
| 8 | 7.918 |

^(a)^Protons are named according to Figure 1A. H^N^ refers to the resonance of the proton bound to the nitrogen.
