# Supplementary material for: Direct and Allosteric Inhibition of the FGF2/HSPGs/FGFR1 Ternary Complex Formation by an Antiangiogenic, Thrombospondin-1-Mimic Small Molecule
Source: PLoS One. 2012 May 14;7(5):e36990. doi: 10.1371/journal.pone.0036990 (PMC3351436; doi:10.1371/journal.pone.0036990)
Supplement: Table S3 — Statistics of the top three FGF2-sm27 clusters obtained with HADDOCK. (DOC) [file pone.0036990.s010.doc]

**Table S3. Statistics of the top three FGF2-sm27 clusters obtained with HADDOCK.**

| **Cluster** | **Haddock score** | **RMSD-E_min_^a^** | **N^b^** | **E_vdw_^c^** | **E_elec_^c^** | **BSA^d^ (Å^2^)** | **E_desolv_^e^** |
| --- | --- | --- | --- | --- | --- | --- | --- |
| 1 | -233.06 ± 28.51 | 1.63 ± 0.13 | 82 | -18.1 ± 3.0 | -217.1 ± 28.7 | 609.24± 21.5 | 1.32 ± 4.59 |
| 2 | -198.49 ± 8.41 | 1.59 ±0.06 | 21 | -26.0 ± 2.0 | -172.5 ± 9.5 | 622.1 ± 3.7 | 0.16 ± 3.46 |
| 3 | -215.12 ± 19.22 | 1.63 ± 0.13 | 16 | -23.2 ± 1.3 | -192.2 ± 18.8 | 628.7 ± 20.0 | -0.25 ± 9.14 |

^a^Overall backbone RMSD from the lowest energy structure.

^b^Number of structures in a given cluster.

^c^The intermolecular energies (kcal mol^-1^) were calculated with the OPLS parameters using a 8.5 Å cut-off.

^d^Buried surface area (Å^2^).

^e^The desolvatation energy (kcal mol^-1^) was calculated using the atomic desolvation parameters by Fernandez-Recio et al. (Fernandez-Recio, J., Totrov, M., and Abagyan, R. (2004) Journal of Molecular Biology 335, 843-865).
