# Supplementary material for: Direct and Allosteric Inhibition of the FGF2/HSPGs/FGFR1 Ternary Complex Formation by an Antiangiogenic, Thrombospondin-1-Mimic Small Molecule
Source: PLoS One. 2012 May 14;7(5):e36990. doi: 10.1371/journal.pone.0036990 (PMC3351436; doi:10.1371/journal.pone.0036990)
Supplement: Table S4 — Average NMR relaxation parameters values of apo- and holo-FGF2 calculated over the residue range 30–152. (DOC) [file pone.0036990.s011.doc]

**Table S4.** Average NMR relaxation parameters values of apo- and holo-FGF2 calculated over the residue range 30-152.

|  | **FGF2 Apo** | **FGF2:sm27 1:2** |
| --- | --- | --- |
| R1 (s^-1^) | 1.89 ± 0.13 | 1.97 ± 0.13 |
| R2 (s^-1^) | 12.51 ± 1.24 | 13.46 ± 1.49 |
| R1rho (s^-1^) | 12.26 ± 1.19 | 11.53 ± 1.07 |
| ^1^H-^15^N NOE | 0.77 ± 0.05 | 0.76 ± 0.06 |
