# Supplementary material for: Direct and Allosteric Inhibition of the FGF2/HSPGs/FGFR1 Ternary Complex Formation by an Antiangiogenic, Thrombospondin-1-Mimic Small Molecule
Source: PLoS One. 2012 May 14;7(5):e36990. doi: 10.1371/journal.pone.0036990 (PMC3351436; doi:10.1371/journal.pone.0036990)
Supplement: Information S1 — Full details of the experimental procedures. (DOC) [file pone.0036990.s012.doc]

**Information S1**

*Chemicals.* Sm27 (NSC37204) was from the Developmental Therapeutics Program, NCI, National Institutes of Health (Rockville, MD). Human recombinant FGF2 was either produced and purified as described [1], or obtained through the NCI-Biological Resources Branch (Frederick, MD). The uniformly ^15^N-labelled 154-residue form of Glu^3,5^ Ser^78,96^ human recombinant FGF2 was produced and purified by ASLA (Riga, Latvia) according to the protocol previously optimized [2,3]. Unfractionated heparin (13.6 kDa) was from Glycores 2000 (Milan, Italy). sFGFR1α(IIIc)/Fc (FGFR1α), was from ReliaTech. Recombinant mouse noggin-Fc was from R&D Systems, Inc.

*FGF2/sm27 interactions assessed by NMR*. NMR samples contained 0.57 mM ^15^N-labeled FGF2, dissolved in a buffer containing 50 mM potassium phosphate, 2 mM NaN_3_, and 10 mM deuterated DTT in 90% H_2_O/10% D_2_O, pH 5.5. For the identification of the binding region, unlabeled sm27 (stock solution 0.27 M) was incrementally added from 0 to 0.57 mM, in a nineteen steps titration (the ligand/protein molar ratio ranged between 0-4). At molar ratios higher than 1:1, partial sample precipitation was observed, till full sample precipitation at nominal 4:1 ratio. Nevertheless, the soluble fraction was folded and the chemical shift variations of the residues involved in binding were progressively observed. Due to the precipitation it was not possible to reach the plateau of the titration and to estimate the real stoichiometric ratio at each step. Freshly prepared samples with 1:1, and 2:1 sm27:FGF2 ratios didn’t show significant precipitate, and the chemical shift variations were comparable to those obtained in the 19 steps titration (data not shown).

NMR data were collected on a Bruker DMX 500 MHz spectrometer. Typically a sweep width of 13 ppm and 40 ppm, 1k x 128 data points were used in proton and nitrogen dimensions, respectively, for the 2D [^1^H-^15^N] HSQC spectra. Experiments were performed at 298 K. Data acquired and processed using Topspin (Bruker Biospin) were apodized with a squared sinebell shifted by 90° and polynomial baseline correction. The analysis was performed by means of Sparky (T. D. Goddard and D. G. Kneller, SPARKY3, University of California, San Francisco).

Chemical shift perturbations were calculated as a weighted sum of ^1^H and ^15^N chemical shift changes [4]. The peaks in the HSQC spectra of the protein, recorded as a function of sm27 additions, were integrated by Sparky software. The intensities of peaks belonging to each spectrum were normalized dividing their intensity by the total intensity, that is the sum of all the measured cross peaks.

A set of standard 2D TOCSY and ROESY experiments were recorded on sm27 to attribute its proton resonances (Table S4). On the FGF2:sm27 1:2 sample a 3D HSQC-NOESY experiment was recorded with a mixing time of 120 ms.

*Model of the FGF2/sm27 complex through HADDOCK calculations.* Molecular docking of sm27 on FGF2 was performed using the software HADDOCK2.0 [5,6]. The bundle of the ten best NMR structures of deposited 1BLD.pdb was used for docking simulation [7]. Ambiguous interaction restraints (AIR) were introduced on the basis of NMR chemical shift perturbation (CSP) data. Residues showing significant CSP upon binding were defined as active and their neighbors were defined as “passive” (Table S1), according to HADDOCK definition. NOE contacts observed in the 3D HSQC-NOESY spectrum of the FGF2:sm27 1:2 sample were converted into distances and used as unambiguous restrains. The geometric coordinates and parameters for the ligand were calculated using the PROGDG server [8], and then manually optimized. Based on ^1^H-^15^N long-range HSQC spectra results, Nε2 protonated tautomer for the three histidines (H25, H44, and H59) was selected in the input files. Initially, 2000 structures were calculated during the rigid docking, and the best 200 solutions based on intermolecular energy were selected for semi-flexible simulated annealing, followed by explicit water refinement. Docking solutions were clustered with 0.8 Å cut off and a minimum of 10 structures per cluster, resulting in 6 clusters ranked according to the average HADDOCK score of their top 5 members. The most populated cluster contained 41% of the structures (82 of 200), had no AIR violations > 0.5 Å, and presented the lowest average energy. The protein-ligand contacts were analyzed using LIGPLOT4.5.3 program [9].

*^15^N relaxation experiments.* HSQC-based experiments were recorded to measure ^15^N longitudinal T1 and transverse T2 relaxation rates [10,11]. Data-sets were collected with 64 and 96 transients per increment for T1 and T2, respectively, using a steady-state recovery time of 3 s. The T1 values were obtained using delays of 20 (x2 times), 60, 140, 240, 360, 520, 720, and 1500 ms. T2 experiments were recorded using delays of 15.3, 30.7, 46.0 (x2 times), 61.3, 76.6, 122.6, 153.3, and 183.9 ms.

For heteronuclear ^1^H→^15^N NOE experiments, interleaved proton-presaturated and non presaturated spectra were acquired. The interleaved spectra were separated by a Bruker standard macro. In the NOE experiment the recycle delay was extended to 4 s. The 2D spectra were recorded in phase-sensitive mode using time proportional incrementation [12] or the echo-antiecho method [13].

The ^15^N rotating frame relaxation rates T1rho were measured using an on resonance rotating frame experiment [14,15]. T1rho values were determined using spectra recorded with delays of 4, 8, 24, 64, 100, 128, 164, 256, and 300 ms. A relaxation delay of 3 s between scans was employed.

Relaxation data were obtained for 131 residues, out of the 146 possible correlations (FGF2 sequence contains 9 prolines) involving backbone amide protons. Resonance overlap was the major cause of difficulties in measuring peak intensities for the remaining 15 residues (M1, A2, F21, H25, L41, V49, Q65, G70, V71, S73, L91, V97, T121, W123, and T130**).**

*NMR Relaxation data processing and analysis.* All spectra were processed with nmrPipe [16], intensities were calculated with nmrView (One Moon Scientific Inc). R1 and R2 relaxation rates were determined by fitting peak intensities to single-exponential two parameter decay curves using the Rate Analysis tool inside nmrView. The Monte Carlo procedure was used to estimate the standard deviation of the data intensities. For the analysis of the relaxation parameters the program Modelfree 4.20 by Palmer and co-workers [17] was used. Estimates of local correlation times for the NH vector of each residue were derived by analyzing R2/R1 ratios using r2r1_tm inside the program quadratic_diffusion (A.G. Palmer, Columbia University, New York). The overall correlation times were calculated as the average over the specific τ_m_ values by selecting only residues in β-strand elements, and excluding residues with steady-state 15N-1H NOE≤ 0.6 and |(R2/R1) − 〈R2/R1〉| ≥ SD [10]. Similar correlation times were estimated from relaxation parameters for the apo (9.2±0.5 ns) and the holo protein (9.5±0.4 ns).

The relaxation data were analyzed with both isotropic and axially symmetric model and the latter model was selected based on the obtained results. Estimates for the rotational diffusion tensor and D_║_/D_┴_ were obtained using two programs: pdbinertia and quadratic_diffusion, both provided on A.G. Palmer’s Web site (A.G. Palmer, Columbia University, New York). The ratios of three principal moments of the inertial tensor of FGF2 were estimated from its NMR solution structure (PDB-code 1BLA, [2]) to be 1.0:0.9:0.78. Using the calculated rotational diffusion tensors, backbone relaxation data were fit to the five standard Lipari–Szabo model-free formalism models [17]. Parameters of the model-free formalism were optimized for each residue individually, and the best parameter set identified by model selection according to d'Auvergne and Gooley [18] and Wright and co-workers [19] with Bayesian Information Criteria (BIC).

*NMR Hydration studies.* 2D ePHOGSY-HSQC experiments with NOE and ROE steps were recorded in order to discriminate between Overhauser and exchange effects. 2D ePHOGSY, followed by ^1^H-^15^N HSQC detection [20] were acquired, combined with WATERGATE pulse sequence for water suppression. The pulse used for selective water excitation was a 50 ms long 180° Gaussian pulse. The mixing and spin-lock periods for NOE and ROE steps, respectively, were 80 ms long. The relative intensities measured in 2D ePHOGSY-NOE and ePHOGSY-ROE of FGF2 in the absence and in the presence of sm27 are reported in Figure S4A, B, respectively. The ratio of normalized intensities in the holo and apo states measured from 2D ePHOGSY-NOE and ROE spectra are reported in Figure S4C.

*Molecular Dynamics studies of the apo FGF2 and FGF2/sm27 complex.* Apo and holo structures were subjected to MD simulation analysis. All MD simulations were performed using the AMBER 9.0 package [21] with the ff03 force field. In each run, the protein was solvated in a cubic box large enough to contain 0.8 nm of solvent around the complex. The TIP3P water model was used for salvation [22]. A 1 nm non-bonded cutoff was used for van der Waals interactions, while the Particle Mesh Ewald summation method (PME) was used to deal with long-range Coulomb interactions [23]. The Berendsen thermostat was used to control temperature and pressure [24]. Charges on sidechains were chosen to correspond to a pH value of 7. Cl^−^ counterions were added to ensure electroneutrality. All the structural and dynamical analyses were performed with in-house built software or with analyses programs from the GROMACS package, after the trajectories were translated to the suitable format.

Before starting production runs, every complex was initially minimized in vacuo by multiple minimizations (200 steps steepest descent plus 200 steps conjugate gradient). After this, each system was solvated. Multiple solute equilibration processes were performed, in order to reorganize the water molecules (100 steps steepest descent + 50 ps equilibration dynamics at constant P and T =100K). After the solute equilibration, another system minimization was performed (200 steps, steepest descent); afterwards, the temperature of the system was slowly brought to the desired value of 300K in 3 steps, with subsequent 100ps NVT equilibration processes at 100K, 200K and 300K). Finally, a last 100 ps equilibration NPT process was performed. From this final structure, a set of 2 different 50ns MD simulations was performed for each system. Two different sets of initial velocities obtained from a Maxwellian velocity distribution at the desired temperature of 300K were used to yield three different production runs for each complex.

*Analysis of ligand-dependent dynamic properties from MD trajectories.* For each MD trajectory we computed on the time interval 25-50 ns, the matrix of distance fluctuations *A,* whose elements are defined as:

(1)

where *d_ij_* is the time-dependent distance of the Cα atoms of amino acids *i* and *j*, and the brackets indicate the time-average over the trajectory. Notice that *A* is invariant under translations and rotations of the molecules and, unlike the covariance matrix, does not depend on the choice of a particular protein reference structure. The *A* matrix, and various parameters derived from it, can be used to characterize the salient elasticity and plasticity properties of a protein undergoing structural fluctuations. The presence of quasi-rigid substructures in the complexes of interest should reflect in specific properties of *A*. In fact, pairs of amino acids belonging to the same quasi-rigid substructure should be associated with much smaller distance fluctuations than amino acid pairs in different domains [25,26]. Consistently with the above observation, the regions in which the geometric strain accumulates can be characterized by using the strain profile introduced in ref. [25,26]. The strain of a given amino acid, *i*, is defined as

(2)

where *j* runs over all protein amino acids, and *f* is a sigmoidal function that restricts the contribution to the sum to amino acids that are within about 5 Å from amino acid *i*: , where *x* is expressed in Ångstroms. Regions that respond differently to the presence of a certain ligand, will be characterized by different values of *p* because in the course of the dynamical evolution their local network of contacts appreciably changes due to the relative motion of neighboring substructures.

*Surface plasmon resonance (SPR) analysis.* SPR measurements were performed on a BIAcore X instrument (GE-Healthcare, WI). Heparin was immobilized onto the SPR chip as already described [27]. Briefly, a CMD50L sensorchip (Xantec Bioanalytics, Dusseldorf, Germany) activated with 50 μl of a mixture containing 0.2 M EDC and 0.5 M NHS was coated with streptavidin. Heparin was biotinylated at its reducing end and immobilized onto the streptavidin-coated sensorchip, allowing the immobilization of 80 resonance units (RU) (6.0 fmol/mm^2^ of heparin). A streptavidin-coated sensorchip was used as reference and for blank subtraction. For the immobilization of FGFR1, the protein (100 ng/mL in 10 mM sodium acetate pH 4.0) was injected onto an activated CMD500L sensorchip (Xantec Bioanalytics) at a flow rate equal to 10 μl/min for 4 min, allowing the immobilization of approximately 3,800 RU (42.0 fmol/mm^2^ of FGFR1). Similar results were obtained for the immobilization of FGF-unrelated noggin protein, here used as a negative control and for blank subtraction. For competition experiments, FGF2 (150 nM) in the absence or in the presence of increasing dilutions of sm27 in 10 mM HEPES, 150 mM NaCl, 3.4 mM EDTA, 0.005% surfactant P20, pH 7.4 (HBS) was injected over the heparin of FGFR1 surfaces for 5 min (to allow the association of the growth factor with the receptors) and then washed until dissociation was observed. After every run, the sensorchip was regenerated by injection of HBS containing 2.0 M NaCl.

*Cell cultures.* Wild-type CHO-K1 cells and the derived HSPG-deficient A745 CHO cell mutants [28], kindly provided by J. D. Esko (La Jolla, CA), were grown in Ham's F-12 medium supplemented with 10% fetal calf serum (FCS). Wild-type CHO-K1 cells do not express significant amount of FGFR1 while FGFR1-transfected A745 CHO flg-1A cells, generated in our laboratory by transfection with the IIIc variant of murine FGFR1 cDNA [29], bear about 30,000 FGFR1 molecules/cell. Bovine aortic endothelial cells (BAEC) [30]were cultured in DMEM supplemented with 10% FCS.

*Binding of FGF2 to cells.* Europium-labeled FGF2 (Eu-FGF2) was prepared as previously described [31]. Endothelial cells (BAEC), naturally expressing both HSPGs and FGFR1, CHO-K1 and FGFR1-expressing A745 CHO cells were used. Subconfluent cell cultures in 96-well plates were washed and incubated for 30 min at 277K in serum-free DMEM with 0.15% gelatin and 25mM HEPES (DMEM-gelatin). The medium was then replaced with cold DMEM-gelatin containing Eu-labeled FGF2 (final concentration 10 ng/ml) with or without the indicated concentrations of sm27, heparin or unlabeled FGF2. The plate was incubated for 2 h at 277K. Wells were washed with cold DMEM-gelatin to remove unbound FGF2, and the amount of total bound FGF2 was detected by adding DELFIA® Enhancement Solution (100 µl, PerkinElmer), and measuring time resolved fluorescence using a Victor^3^ multilabel plate reader (PerkinElmer), as described [31]. In order to determine the amount of FGF2 bound to high affinity receptors on endothelial cells, BAEC were washed with cold 2 M NaCl in 20 mM HEPES (pH 7.5) to remove FGF2 bound to low affinity HSPGs [32], allowing to measure the remaining FGFR1-bound FGF2, detected as indicated above.

*FGF2-mediated cell-cell adhesion assay*: This assay was performed as described previously [33], with minor modifications. Briefly, monolayers of wild-type CHO-K1 cells were washed with PBS and fixed with 3% glutaraldehyde in PBS for 2 h at 277K followed by washing with 0.1 M glycine, and PBS. Then, A745 CHO flg-1A cells (50,000 cells/cm^2^) were added to CHO-K1 monolayers in serum-free medium plus 10 mM EDTA with or without FGF2 (1.66 nM) in the presence of increasing concentrations of sm27. After 2 h of incubation at 310K, unattached cells were removed by PBS washing and A745 CHO flg-1A cells bound to the CHO-K1 monolayer were counted under an inverted microscope at ×125 magnification. Data are expressed as the mean of the cell counts of three microscopic fields chosen at random. All experiments were performed in triplicate and repeated twice.

**References**

1. Isacchi A, Statuto M, Chiesa R, Bergonzoni L, Rusnati M, et al. (1991) A six-amino acid deletion in basic fibroblast growth factor dissociates its mitogenic activity from its plasminogen activator-inducing capacity. Proc Natl Acad Sci U S A 88: 2628-2632.

**2. Moy FJ, Seddon AP, Campbell EB, Bohlen P, Powers R (1995) 1H, 15N, 13C and 13CO assignments and secondary structure determination of basic fibroblast growth factor using 3D heteronuclear NMR spectroscopy. J Biomol NMR 6: 245-254.**

**3. Seddon A, Decker M, Muller T, Armellino D, Kovesdi I, et al. (1991) Structure/activity relationships in basic FGF. Ann N Y Acad Sci 638: 98-108.**

**4. Mulder FA, Schipper D, Bott R, Boelens R (1999) Altered flexibility in the substrate-binding site of related native and engineered high-alkaline Bacillus subtilisins. J Mol Biol 292: 111-123.**

**5. de Vries SJ, van Dijk AD, Krzeminski M, van Dijk M, Thureau A, et al. (2007) HADDOCK versus HADDOCK: new features and performance of HADDOCK2.0 on the CAPRI targets. Proteins 69: 726-733.**

**6. Dominguez C, Boelens R, Bonvin AM (2003) HADDOCK: a protein-protein docking approach based on biochemical or biophysical information. J Am Chem Soc 125: 1731-1737.**

**7. Moy FJ, Seddon AP, Bohlen P, Powers R (1996) High-resolution solution structure of basic fibroblast growth factor determined by multidimensional heteronuclear magnetic resonance spectroscopy. Biochemistry 35: 13552-13561.**

**8. Schüttelkopf AW, Van Aalten DMF (2004) PRODRG: a tool for high-throughput crystallography of protein–ligand complexes. Acta Crystallographica Section D 60: 1355-1363.**

**9. Wallace AC, Laskowski RA, Thornton JM (1995) LIGPLOT: a program to generate schematic diagrams of protein-ligand interactions. Protein Eng 8: 127-134.**

**10. Kay LE, Torchia DA, Bax A (1989) Backbone dynamics of proteins as studied by 15N inverse detected heteronuclear NMR spectroscopy: application to staphylococcal nuclease. Biochemistry 28: 8972-8979.**

**11. Stone MJ, Fairbrother WJ, Palmer AG, 3rd, Reizer J, Saier MH, Jr., et al. (1992) Backbone dynamics of the Bacillus subtilis glucose permease IIA domain determined from 15N NMR relaxation measurements. Biochemistry 31: 4394-4406.**

**12. Marion D, Wuthrich K (1983) Application of phase sensitive two-dimensional correlated spectroscopy (COSY) for measurements of 1H-1H spin-spin coupling constants in proteins. Biochem Biophys Res Commun 113: 967-974.**

**13. Keeler J, Clowes RT, Davis AL, Laue ED (1994) Pulsed-field gradients: theory and practice. Methods Enzymol 239: 145-207.**

**14. Mulder FAA, de Graaf RA, Kaptein R, Boelens R (1998) An Off-resonance Rotating Frame Relaxation Experiment for the Investigation of Macromolecular Dynamics Using Adiabatic Rotations. J Magn Reson 131: 351-357.**

**15. Dayie KT, Wagner G (1994) Relaxation-Rate Measurements for 15Nâˆ’1H Groups with Pulsed-Field Gradients and Preservation of Coherence Pathways. Journal of Magnetic Resonance, Series A 111: 121-126.**

**16. Delaglio F, Grzesiek S, Vuister GW, Zhu G, Pfeifer J, et al. (1995) NMRPipe: a multidimensional spectral processing system based on UNIX pipes. J Biomol NMR 6: 277-293.**

**17. Mandel AM, Akke M, Palmer AG, 3rd (1995) Backbone dynamics of Escherichia coli ribonuclease HI: correlations with structure and function in an active enzyme. J Mol Biol 246: 144-163.**

**18. d'Auvergne EJ, Gooley PR (2003) The use of model selection in the model-free analysis of protein dynamics. J Biomol NMR 25: 25-39.**

**19. Chen J, Brooks CL, 3rd, Wright PE (2004) Model-free analysis of protein dynamics: assessment of accuracy and model selection protocols based on molecular dynamics simulation. J Biomol NMR 29: 243-257.**

**20. Dalvit C, Hommel U (1995) Sensitivity-Improved Detection of Protein Hydration and Its Extension to the Assignment of Fast-Exchanging Resonances. Journal of Magnetic Resonance, Series B 109: 334-338.**

**21. Case DA, Darden TA, Cheatham TEI, Simmerling CL, Wang J, et al. (2006) AMBER 9. University of California, San Francisco.**

**22. Jorgensen WL, Chandrasekhar J, Madura JD, Impey RW, Klein ML (1983) Comparison of simple potential functions for simulating liquid water. J Chem Phys 79: 926-935.**

**23. Darden T, York D, Pedersen L (1993) Particle Mesh Ewald-an N.Log(N) method for Ewald sums in large systems. J Chem Phys 98: 10089-10092.**

**24. Berendsen HJC, Postma JPM, van Gunsteren WF, DiNola A, Haak JR (1984) Molecular dynamics with coupling to an external bath. The Journal of Chemical Physics 81: 3684-3690.**

**25. Aleksiev T, Potestio R, Pontiggia F, Cozzini S, Micheletti C (2009) PiSQRD: a web server for decomposing proteins into quasi-rigid dynamical domains. Bioinformatics 25: 2743-2744.**

**26. Potestio R, Pontiggia F, Micheletti C (2009) Coarse-grained description of protein internal dynamics: an optimal strategy for decomposing proteins in rigid subunits. Biophys J 96: 4993-5002.**

**27. Rusnati M, Urbinati C, Caputo A, Possati L, Lortat-Jacob H, et al. (2001) Pentosan polysulfate as an inhibitor of extracellular HIV-1 Tat. J Biol Chem 276: 22420-22425.**

**28. Esko JD (1991) Genetic analysis of proteoglycan structure, function and metabolism. Curr Opin Cell Biol 3: 805-816.**

**29. Liekens S, Leali D, Neyts J, Esnouf R, Rusnati M, et al. (1999) Modulation of fibroblast growth factor-2 receptor binding, signaling, and mitogenic activity by heparin-mimicking polysulfonated compounds. Mol Pharmacol 56: 204-213.**

**30. Taraboletti G, Belotti D, Dejana E, Mantovani A, Giavazzi R (1993) Endothelial cell migration and invasiveness are induced by a soluble factor produced by murine endothelioma cells transformed by polyoma virus middle T oncogene. Cancer Res 53: 3812-3816.**

**31. Colombo G, Margosio B, Ragona L, Neves M, Bonifacio S, et al. (2010) Non-peptidic thrombospondin-1 mimics as fibroblast growth factor-2 inhibitors: an integrated strategy for the development of new antiangiogenic compounds. J Biol Chem 285: 8733-8742.**

**32. Moscatelli D (1988) Metabolism of receptor-bound and matrix-bound basic fibroblast growth factor by bovine capillary endothelial cells. J Cell Biol 107: 753-759.**

**33. Leali D, Belleri M, Urbinati C, Coltrini D, Oreste P, et al. (2001) Fibroblast growth factor-2 antagonist activity and angiostatic capacity of sulfated Escherichia coli K5 polysaccharide derivatives. J Biol Chem 276: 37900-37908.**
